# The Oxidative Stress-Induced Hypothetical Protein PG_0686 in Porphyromonas gingivalis W83 Is a Novel Diguanylate Cyclase

**DOI:** 10.1128/spectrum.04411-22

**Published:** 2023-01-31

**Authors:** Alexia D. Ximinies, Yuetan Dou, Arunima Mishra, Kangling Zhang, Champion Deivanayagam, Charles Wang, Hansel M. Fletcher

**Affiliations:** a Division of Microbiology & Molecular Genetics, School of Medicine, Loma Linda University, Loma Linda, California, USA; b Department of Pharmacology, University of Texas Medical Branch, Galveston, Texas, USA; c Department of Biochemistry and Molecular Genetics, University of Alabama, Birmingham, Alabama, USA; University of Florida

**Keywords:** PG_0686, *Porphyromonas gingivalis*, c-di GMP, diguanylate cyclase, oxidative stress

## Abstract

The survival/adaptation of Porphyromonas gingivalis to the inflammatory environment of the periodontal pocket requires an ability to overcome oxidative stress. Several functional classes of genes, depending on the severity and duration of the exposure, were induced in P. gingivalis under H_2_O_2_-induced oxidative stress. The *PG_0686* gene was highly upregulated under prolonged oxidative stress. PG_0686, annotated as a hypothetical protein of unknown function, is a 60 kDa protein that carries several domains including hemerythrin, PAS10, and domain of unknown function (DUF)-1858. Although PG_0686 showed some relatedness to several diguanylate cyclases (DGCs), it is missing the classical conserved, active site sequence motif (GGD[/E]EF), commonly observed in other bacteria. PG_0686-related proteins are observed in other anaerobic bacterial species. The isogenic mutant P. gingivalis FLL361 (Δ*PG_0686*::*ermF*) showed increased sensitivity to H_2_O_2_, and decreased gingipain activity compared to the parental strain. Transcriptome analysis of P. gingivalis FLL361 showed the dysregulation of several gene clusters/operons, known oxidative stress resistance genes, and transcriptional regulators, including PG_2212, CdhR and PG_1181 that were upregulated under normal anaerobic conditions. The intracellular level of c-di-GMP in P. gingivalis FLL361 was significantly decreased compared to the parental strain. The purified recombinant PG_0686 (rPG_0686) protein catalyzed the formation of c-di-GMP from GTP. Collectively, our data suggest a global regulatory property for PG_0686 that may be part of an unconventional second messenger signaling system in P. gingivalis. Moreover, it may coordinately regulate a pathway(s) vital for protection against environmental stress, and is significant in the pathogenicity of P. gingivalis and other anaerobes.

**IMPORTANCE** Porphyromonas gingivalis is an important etiological agent in periodontitis and other systemic diseases. There is still a gap in our understanding of the mechanisms that P. gingivalis uses to survive the inflammatory microenvironment of the periodontal pocket. The hypothetical *PG_0686* gene was highly upregulated under prolonged oxidative stress. Although the tertiary structure of PG_0686 showed little relatedness to previously characterized diguanylate cyclases (DGCs), and does not contain the conserved GGD(/E)EF catalytic domain motif sequence, an ability to catalyze the formation of c-di-GMP from GTP is demonstrated. The second messenger pathway for c-di-GMP was previously predicted to be absent in P. gingivalis. PG_0686 paralogs are identified in other anaerobic bacteria. Thus, PG_0686 may represent a novel class of DGCs, which is yet to be characterized. In conclusion, we have shown, for the first time, evidence for the presence of c-di-GMP signaling with environmental stress protective function in P. gingivalis.

## INTRODUCTION

Porphyromonas gingivalis, a black-pigmented, Gram-negative anaerobe, is an important etiological agent of periodontal disease, and is also linked to multiple systemic conditions including cancer (1, 2), cardiovascular disease (2, 3), rheumatoid arthritis (4–7), and Alzheimer’s disease (8–11) . P. gingivalis, as a “keystone pathogen” (12), is further highlighted by its ability to adapt to the harsh inflammatory conditions of the periodontal pocket and disrupt host-microbial homeostasis, which is partly responsible for the pathology observed in periodontal disease, suggesting that P. gingivalis may utilize multiple strategies to survive environmental stress, and orchestrate the microbial/host activities that can lead to disease. Because the environmental stress response in the pathogen is a major determinant of its virulence, a comprehensive understanding of its survival strategies is vital.

Bacterial protective mechanisms against specific oxidants involve multiple systems to sense and rapidly respond to their changing levels in both the cellular and extracellular environments (13). As such, overcoming oxidative stress is a key element in the survival of P. gingivalis in the inflammatory environment of the periodontal pocket. Several systems, including antioxidant enzymes, DNA-binding proteins, the hemin layer, and enzymatic removal of deleterious products (e.g., 8-oxoG, caused by reactive oxygen species), can defend and protect P. gingivalis against oxidative stress-induced damage (reviewed in 13). There is also emerging evidence that suggests the extracytoplasmic function (ECF) sigma factors PG_1660 and PG_1827 could be involved in the regulation of a yet-to-be defined oxidative stress resistance pathway in P. gingivalis (14). Collectively, this continues to raise the question of a complex oxidative stress resistance mechanism in P. gingivalis with likely novel and/or redundant pathways. While most of these responses to oxidative stress may involve transcriptional changes mediated by oxidative modification of specific transcriptional regulators in P. gingivalis, there is still a gap in our understanding of their mechanisms and the involvement of other regulatory systems. Moreover, the integration of functionally divergent effectors/regulators into a regulatory pathway for oxidative/environmental stress adaptation is unclear.

Previously, our lab assessed the transcriptome response of P. gingivalis to conditions of oxidative stress, depending on the duration or level of exposure (15). Collectively, our data indicated that P. gingivalis has an adaptive response to oxidative stress which may involve several previously unrecognized genes (15, 16). Moreover, bacterial cells exposed to a shorter duration of hydrogen peroxide (H_2_O_2_) revealed increased expression of genes involved in DNA repair while after a longer duration, genes involved in protein fate, protein folding, and stabilization were upregulated (15). The gene encoding PG_0686, annotated as a hypothetical protein, was shown to be induced in P. gingivalis under conditions of prolonged (15 min) H_2_O_2_-induced stress (15). In other reports, its induction in P. gingivalis is also observed in the presence of nitric oxide (NO) donor diethylamine nonoate (DEA NONOate) (17, 18) or oxygen (O_2_) (16), and is highly upregulated during the contact of the bacterium with epithelial (19) and invasion of endothelial (20) cells.

Diguanylate cyclases (DGCs), which are described as GGD(/E)EF proteins due to their characteristic active site sequence motif, catalyze the formation of bis-(3′,5′)-cyclic dimeric GMP (21, 22). c-di-GMP is a ubiquitous bacterial “second messenger,” regulating processes, such as cell differentiation, cell cycle progression, biofilm formation, motility, oxidative stress resistance, and virulence (23, 24). Together with c-di-GMP, phosphodiesterases (PDE), and the appropriate effectors, these DGC proteins are part of a bacterial signal transduction system which is responsible for sensing environmental signals and adjusting the cellular behavior and/or metabolism in response to these signals (23–26). While c-di-GMP as a second messenger has been known for more than 30 years, its signaling system, which is widely distributed in more than 85% of bacteria, is predicted to be missing or undetected in P. gingivalis (23). Instead, P. gingivalis strains were shown to predominantly synthesize bis-(3′,5′)-cyclic di-AMP (c-di-AMP), in an atypical signaling system that is essential for their growth and survival (27). Another report using bioinformatics tools, however, identified a GGD(/E)EF domain carrying protein in P. gingivalis ATCC 33277 (28). While there was no direct evidence for the function of this protein (PGN_1932) in the catalysis of c-di-GMP from GTP, a *PGN_1932*-defective mutant in P. gingivalis ATCC 33277 showed decreased intracellular c-di-GMP levels, and had a negative impact on biofilm formation and host cell invasion (28). These observations have raised the possibility that other proteins may also be involved in c-di-GMP synthesis in P. gingivalis. It is noteworthy that P. gingivalis W83 carries a similar protein (PG_1987) with 89% identity to PGN_1932, and is annotated as a CRISPR-associated Csm1 family protein.

In this study, we have further characterized the hypothetical protein PG_0686 to elucidate its role in oxidative stress resistance, virulence, and secondary messenger signaling in P. gingivalis. Under conditions of oxidative stress, inactivation of the *PG_0686* gene in P. gingivalis W83 modulated the intracellular level of c-di-GMP, the dysregulation of several transcriptional regulators, and oxidative stress/virulence genes. The purified recombinant P. gingivalis PG_0686 protein, which has features of DGCs but is missing a “GGD(/E)EF” characteristic active site sequence motif, catalyzed the formation of c-di-GMP from GTP. The implication that PG_0686 may play a role as an important global regulator in P. gingivalis is discussed.

## RESULTS

### *PG_0686* was upregulated under multiple environmental stress conditions.

**FIG 1:**
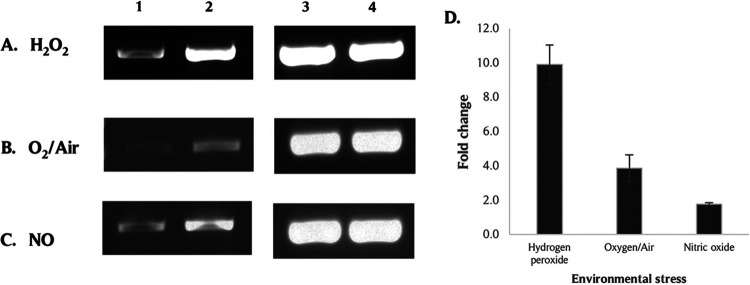
The induction of *PG_0686* gene in P. gingivalis W83 under different types of environmental stress. P. gingivalis W83 cultures were treated with (A) H_2_O_2_ (hydrogen peroxide stress, HPS), (B) O_2_/Air, or (C) NO (from NO donor diethylamine (DEA) NONOate), and total RNA was extracted from the samples and subjected to RT-PCR. (A, B, and C) Lane 1: *PG_0686* gene from untreated control samples; Lane 2: *PG_0686* gene from environmental stress-treated samples; Lane 3: *16S rRNA* gene from untreated control samples; Lane 4: *16S rRNA* gene from environmental stress-treated samples. (D) qRT-PCR confirmed *PG_0686* induction under environmental stress. The results represent 3 independent experiments. Error bars indicate standard error of the mean (SEM). The *PG_0686* gene was induced under multiple types of environmental stress.

To confirm the induction of *PG_0686* in P. gingivalis W83 under different types of environmental stress, reverse transcription - polymerase chain reaction (RT-PCR) and quantitative RT-PCRs (qRT-PCR) were used to evaluate the modulation of this gene when P. gingivalis was exposed to 0.25 mM H_2_O_2_, 13 μM NO for 15 min, or O_2_/air for 1 h, respectively. Compared to normal anaerobic (AN) conditions, the *PG_0686* gene is differentially upregulated in P. gingivalis W83 in the presence of various environmental stresses (Fig. 1). The upregulation of *PG_0686* was most robust when P. gingivalis W83 was treated with H_2_O_2_, with a fold change of 9.90 (±1.14). In the presence of O_2_/air or NO, there was an upregulation of 3.85 (±0.78) and 1.76 (±0.10) fold, respectively.

### The *PG_0686*-deficient P. gingivalis isogenic mutant, FLL361, showed decreased oxidative stress sensitivity and survival.

**FIG 2:**
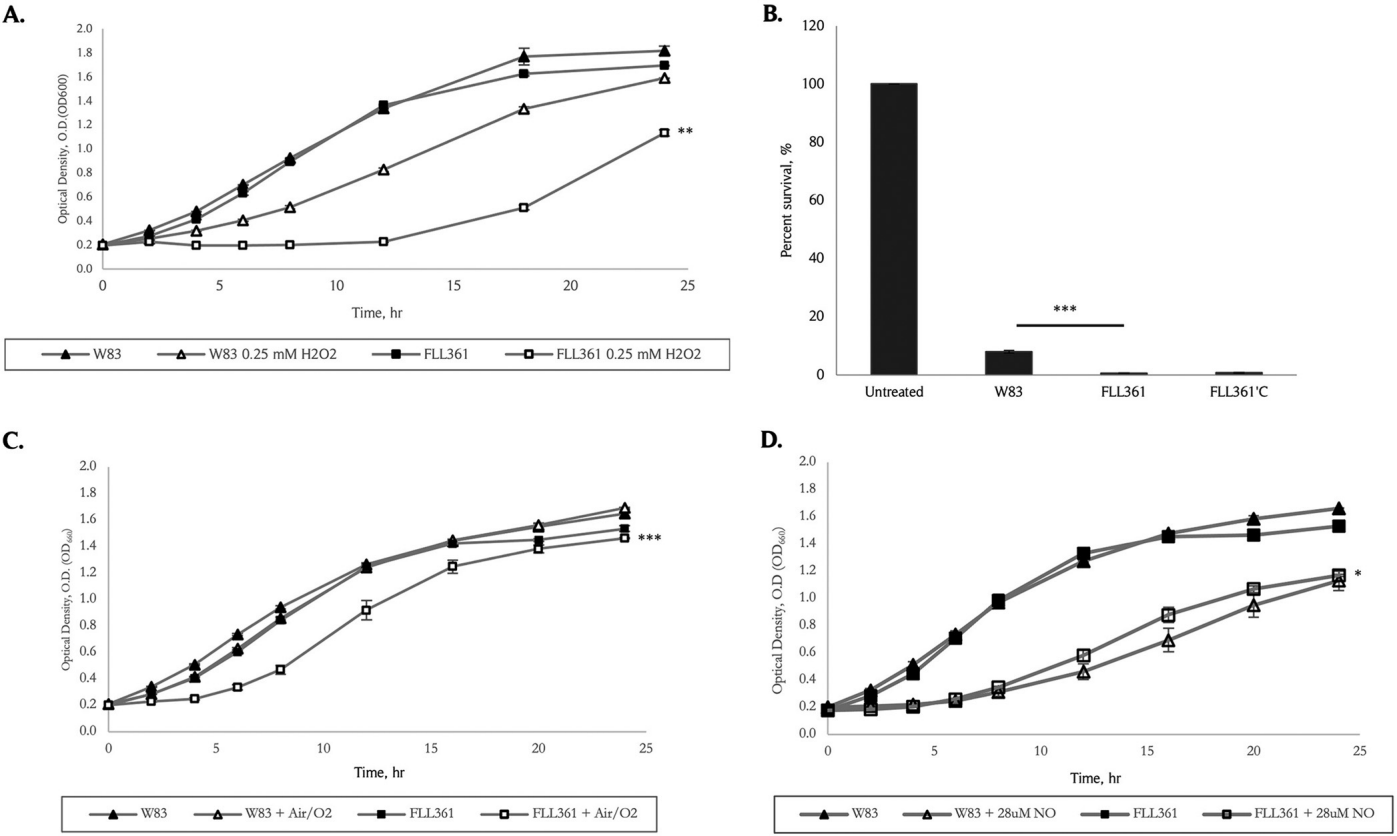
The sensitivity and survival of the P. gingivalis *PG_0686*-deficient mutant, FLL361, under multiple environmental stress, compared to the W83 wild type. Strains were grown in BHI broth, from overnight cultures, to OD_600_ ~ 0.2. The cultures were treated with H_2_O_2_ (HPS), Air/O_2_, or NO (DEA NONOate), and left to grow over a 24-h period. For percent survival, at a 12 h time point, H_2_O_2_-treated culture portions were removed from the broth, diluted, plated on BHI agar plates, and grown 5 to 7 days. Colonies were enumerated and expressed as a percentage of the untreated controls for the respective strains. Untreated controls were included in the experiments, and the results represent 3 independent experiments. (A) W83 and FLL361 sensitivity to HPS. (B) W83 and FLL361 survival in the presence of HPS. (C) W83 and FLL361 sensitivity to O_2_/Air. (D) W83 and FLL361 sensitivity to NO. Compared to W83, the FLL361 mutant was significantly more sensitive to different stresses, except NO (A to D). Error bars indicate standard error of the mean (SEM). (*, *P* < 0.1; **, *P* < 0.05; ***, *P* ≤ 0.001).

To assess the functional role of *PG_0686* in oxidative stress resistance, an isogenic mutant of W83, deficient in this gene, was constructed via allelic-exchange mutagenesis (Fig. S1A). The isogenic mutant, designated P. gingivalis FLL361 (Δ*PG_0686*::*ermF*), was black-pigmented and had beta-hemolytic activity similar to the wild-type (data not shown). The P. gingivalis FLL361 isogenic mutant had a similar initial growth rate to the wild-type W83 strain, but displayed increased sensitivity to H_2_O_2_ (Fig. 2A). Following a 15 min exposure to 0.25 mM H_2_O_2_, the wild-type strain and FLL361 had 7.98% and 0.61% survival rates, respectively. Complementation (in *trans* using plasmid pT-COW) of the mutant strain FLL361 did not restore the wild-type phenotype, as the complemented strain FLL361’C showed a 0.7% survival rate under H_2_O_2_-induced stress (HPS) conditions (Fig. 2B). A RT-PCR analysis showed the expression of the *PG_0686* gene in the complemented FLL361’C strain, however it was not induced under H_2_O_2_ stress-induced conditions (Fig. S1B). Taken together, the data indicate a possible complex regulatory mechanism for gene function. Sensitivity to O_2_/air or NO-induced stress was less pronounced in P. gingivalis FLL361 compared to the wild-type strain (Fig. 2C and D).

### Transcriptome analysis of P. gingivalis FLL361 showed modulation of several important genes.

**FIG 3:**
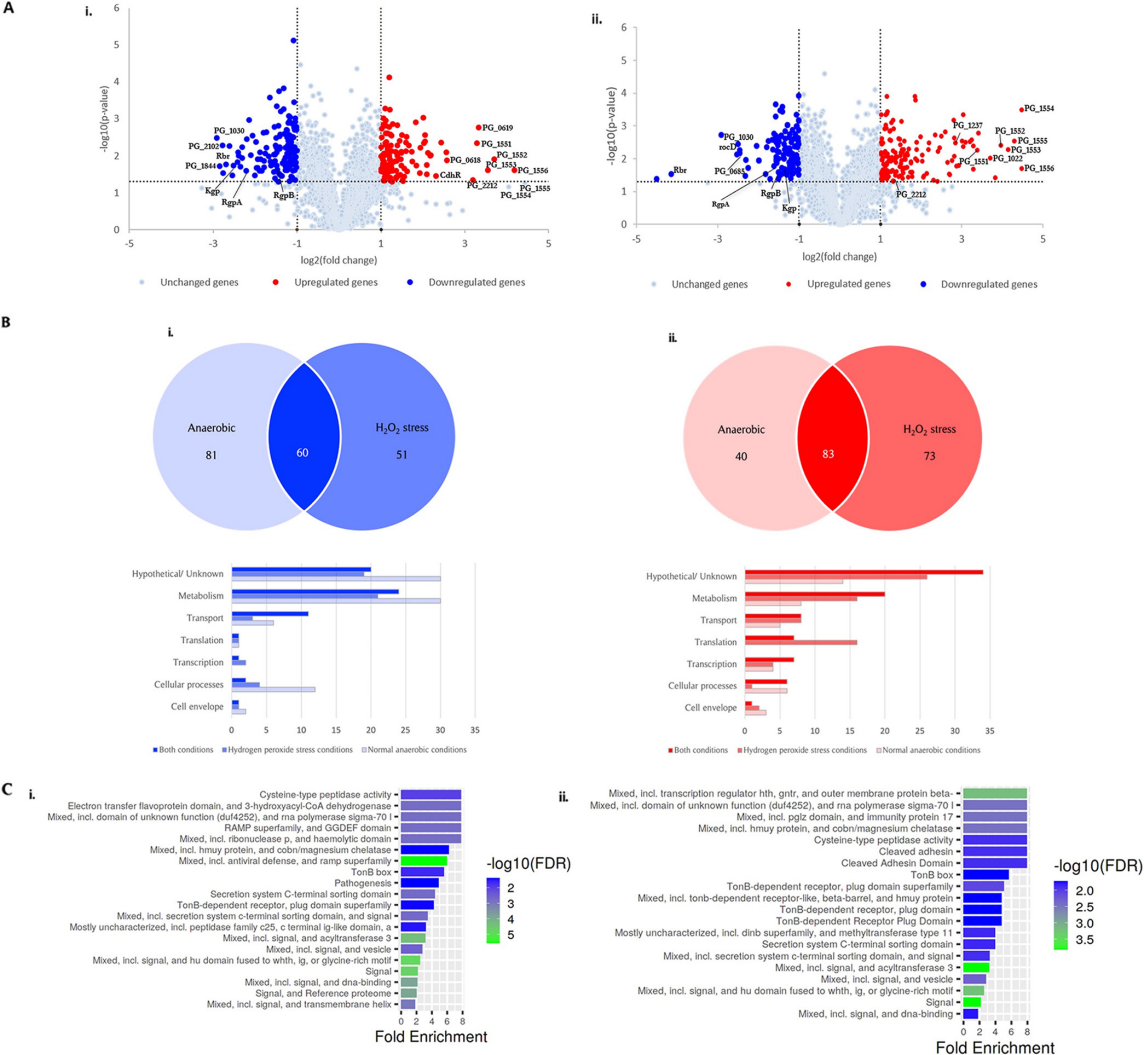
Transcriptome analysis of FLL361. The genes that were differentially expressed were ≥ 2-fold up- or downregulated (*P* ≤ 0.05). (A) Volcano plots of differentially expressed genes between P. gingivalis *PG_0686*-deletion mutant FLL361 and W83 wild-type under: (A, panel i.) AN conditions and (A, panel ii.) HPS conditions. (B) Distribution of functions for P. gingivalis FLL361 genes that were: (B, panel i.) downregulated (blue) or (B, panel ii.) upregulated (red) under AN conditions, HPS conditions, or both conditions. Differentially expressed genes were grouped by functional class according to the KEGG P. gingivalis genome database (https://www.genome.jp/kegg-bin/show_genome?org=pgi). (C) The top enriched pathways in P. gingivalis FLL361 under AN (C, panel i.) and HPS (C, panel ii.) conditions. Enrichment plots were generated by ShinyGO 0.76 (31).

A genome-wide transcriptome analysis by RNA-sequencing (RNA-seq) was performed in order to further evaluate the role of the *PG_0686* gene in HPS resistance in P. gingivalis (Fig. 3A). The RNA-seq gene expression profile was confirmed by qRT-PCR analysis of 10 genes that were observed to be highly regulated in FLL361 (Table S1). Approximately 13% of the genome displayed altered expression more than 2-fold (123 upregulated genes; 141 downregulated genes) under AN conditions (Fig. 3B). Among the differentially expressed genes, 44 were annotated as unknown functional/hypothetical protein, including 14 that were upregulated and 30 that were downregulated. Table 1 lists gene clusters and important genes that were differentially upregulated in FLL361 under both AN and HPS conditions. Of note is the *PG_1551*-*PG_1556* (*hmu*) gene cluster, which was the most highly upregulated (9- to 21-fold). The *hmu* operon is one of 3 multigene clusters encoding genes that have been demonstrated to be involved in the heme acquisition/utilization in P. gingivalis (reviewed in 29). Also upregulated were genes, *PG_0619* (10.08-fold) and *PG_0618* (5.97-fold), encoding the alkyl hydroperoxide reductase. Several transcriptional regulators, including *PG_2212*, *cdhR* (*PG_1237*) and *PG_1181* were upregulated 9.14-, 4.97-, and 4.15-fold, respectively. The gene clusters and important genes that were differentially downregulated in FLL361, under both AN and HPS conditions, are shown in Table 2. The rubrerythrin (*rbr; PG_0195*) and the *PG_1030* genes were the most highly downregulated at 6.50- and 7.57-fold, respectively. *PG_0682*, *PG_0683*, *PG_0684*, and *PG_0685*, encoding ABC transporter system proteins were downregulated more than 2-fold. Gingipain genes *RgpB* (*PG_0506*) and *RgpA* (*PG_2024*) were downregulated 2.79- and 4.62-fold respectively, while *Kgp* (*PG_1844*) was downregulated 7.19-fold and 3.38-fold under AN and HPS conditions, respectively. This downregulation of the gingipains predicts less proteolytic activity and likely reduced virulence in FLL361, when compared to W83. It is noteworthy that the Type IX secretion system (TSS9) genes were downregulated 3- to 7-fold. Under HPS in P. gingivalis FLL361, approximately 156 genes were upregulated and 111 downregulated. Compared to AN conditions, several genes showed slight changes under HPS. The modulation of the *PG_1551-PG_1556* (*hmu*) gene cluster remained unchanged (9- to 21-fold upregulated). Genes encoding for the ECF sigma factor (*PG_0214*) and putative anti-sigma factor (*PG_0215*) were both upregulated approximately 10-fold. The most dramatic change occurred in the *rbr* gene that was downregulated 17.67-fold under HPS conditions compared to 6.50-fold under normal AN conditions (Table 2). The top enriched pathways, as obtained from ShinyGO (30), included signaling, transcription, *hmuY* (haem acquisition), and TonB-dependent receptor function (i.e., transport across the outer membrane) (Fig. 3C). Specifically, the TonB-dependent receptor and ABC transporter transmembrane region are enriched in the differentially upregulated genes, in both AN and HPS conditions (Fig. S2). Conversely, virulence and secretion pathways were enriched in the differentially downregulated genes. Collectively, the data suggest that the *PG_0686* gene may be part of a common mechanism(s) that can modulate multiple genes and their corresponding pathways.

**TABLE 1:** Genes clusters and important genes differentially upregulated in FLL361 under both anaerobic and hydrogen peroxide stress conditions

		AN^*a*^		HPS^*b*^	
Gene ID	Annotation	Fold change	*P* value	Fold change	*P* value
*PG_0199*	TatD family hydrolase	2.60	8.34E-03	2.86	3.31E-02
*PG_0200*	Membrane protein insertion efficiency factor YidD	2.63	1.51E-03	4.17	3.56E-02
*PG_0201*	Ribonuclease P protein component	2.36	5.14E-03	3.97	2.10E-03
*PG_0202*	Uroporphyrinogen-III synthase	2.22	2.43E-03	2.54	3.22E-02
*PG_0214*	Sigma-70 family RNA polymerase sigma factor	3.03	7.37E-03	9.71	2.10E-02
*PG_0215*	Hypothetical protein	2.56	3.82E-02	10.62	1.64E-03
*PG_0216*	DUF4252 domain-containing protein	2.39	7.59E-03	8.22	4.31E-03
*PG_0217*	Hypothetical protein	2.94	2.18E-02	6.80	1.15E-02
*PG_0218*	Hypothetical protein	2.63	1.00E-02	5.34	5.87E-03
*PG_0615*	Translational GTPase TypA	2.30	7.49E-05	2.74	2.38E-03
*PG_0618*	Alkyl hydroperoxide reductase subunit C	5.97	1.31E-02	2.07	9.93E-03
*PG_0619*	Alkyl hydroperoxide reductase subunit F	10.08	1.73E-03	2.43	9.21E-03
*PG_0928*	Two-component system response regulator	2.13	3.78E-02	3.63	9.91E-03
*PG_0929*	Hypothetical protein	2.95	3.62E-02	4.57	3.31E-03
*PG_0930*	Hypothetical protein	3.30	1.11E-01	5.27	4.95E-02
*PG_0931*	DNA-binding protein	2.27	2.64E-02	4.08	1.17E-02
*PG_0985*	Sigma-70 family RNA polymerase sigma factor	2.57	2.56E-02	6.05	1.49E-03
*PG_0986*	Hypothetical protein	2.83	2.70E-03	7.06	2.28E-03
*PG_0987*	DUF4252 domain-containing protein	2.35	1.80E-02	5.63	2.07E-03
*PG_1019*	DUF4876 domain-containing protein	4.03	9.22E-04	5.50	4.54E-03
*PG_1020*	TonB-dependent receptor	4.36	8.33E-03	9.20	2.98E-03
*PG_1178*	Hypothetical protein	4.15	4.06E-03	6.24	1.31E-02
*PG_1180*	Membrane protein	4.05	4.60E-03	8.03	2.93E-03
*PG_1181*	TetR/AcrR family transcriptional regulator	4.15	2.73E-03	8.52	3.13E-03
*PG_1237*	DNA-binding response regulator (CdhR)	4.97	3.54E-02	7.18	3.24E-03
*PG_1551*	Heme-binding protein HmuY	9.76	4.60E-03	9.77	4.09E-03
*PG_1552*	TonB-dependent receptor	13.07	1.23E-02	15.55	3.94E-03
*PG_1553*	Cobaltochelatase	11.76	2.41E-02	17.56	5.26E-03
*PG_1554*	Hypothetical protein	16.48	6.89E-02	22.14	3.25E-04
*PG_1555*	Hypothetical protein	21.60	5.39E-02	19.64	2.92E-03
*PG_1556*	DUF2149 domain-containing protein	18.26	2.41E-02	22.09	2.01E-02
*PG_2212*	Transcriptional regulator	9.14	4.56E-02	2.73	6.03E-02

a AN – anaerobic conditions.

b HPS – hydrogen peroxide stress conditions.

**TABLE 2:** Gene clusters and important genes differentially downregulated in FLL361 under both anaerobic and hydrogen peroxide stress conditions

Gene ID	Annotation	AN^*a*^		HPS^*b*^	
		Fold change	*P* value	Fold change	*P* value
*PG_0001*	Chromosomal replication initiator protein DnaA	−2.12	7.46E-06	−2.55	5.34E-03
*PG_0002*	N-acetyltransferase	−2.00	4.64E-03	−2.03	1.35E-02
*PG_0185*	TonB-dependent receptor	−3.82	1.23E-02	−2.36	6.77E-03
*PG_0186*	RagB/SusD family nutrient uptake outer membrane protein	−4.94	5.87E-03	−2.75	3.34E-04
*PG_0195*	Rubrerythrin	−6.50	1.68E-02	−17.67	2.93E-02
*PG_0506*	Gingipain R2 (RgpB)	−2.79	1.84E-02	−2.76	3.20E-02
*PG_0611*	Lipoprotein	−3.87	2.14E-02	−1.99	2.14E-02
*PG_0612*	Hypothetical protein	−	−	−	−
*PG_0613*	Hypothetical protein	−4.96	1.17E-02	−2.95	8.64E-03
*PG_0614*	Hypothetical protein	−5.25	1.01E-02	−3.16	2.63E-03
*PG_0682*	ABC transporter permease	−1.86	1.66E-01	1.92	2.20E-01
*PG_0683*	ABC transporter permease	−2.72	1.53E-03	1.17	1.91E-01
*PG_0684*	ABC transporter permease	−2.78	2.33E-02	−2.62	7.80E-03
*PG_0685*	ABC transporter ATP-binding protein	−5.81	3.40E-02	−5.49	6.84E-03
*PG_1030*	T9SS C-terminal target domain-containing protein	−7.57	3.26E-03	−7.57	1.85E-03
*PG_1269*	1-pyrroline-5-carboxylate dehydrogenase	−5.33	1.46E-02	−5.75	7.54E-03
*PG_2170*	Amidinotransferase	−1.78	1.80E-02	−2.30	1.33E-02
*PG_2171*	Ornithine-oxo-acid transaminase	−1.74	6.64E-03	−2.02	3.04E-02
*PG_1809*	2-oxoglutarate ferredoxin oxidoreductase subunit gamma	−2.61	6.27E-04	−2.94	1.16E-02
*PG_1810*	2-oxoglutarate oxidoreductase	−2.33	5.27E-03	−2.35	1.70E-02
*PG_1844*	Lysine-specific cysteine protease (Kgp)	−7.19	1.90E-02	−3.38	2.86E-03
*PG_1987*	Type III-B CRISPR-associated protein Cas10/Cmr2	−3.73	2.55E-02	−1.55	3.30E-02
*PG_2024*	Gingipain R2 (RgpA)	−4.62	2.51E-02	−2.84	2.56E-02
*PG_2100*	T9SS C-terminal target domain-containing protein	−2.79	7.90E-03	−3.98	1.13E-02
*PG_2101*	Hypothetical protein	−3.44	1.37E-02	−2.83	4.16E-02
*PG_2102*	T9SS C-terminal target domain-containing protein	−6.86	5.22E-03	−4.94	1.10E-02

a AN – anaerobic conditions.

b HPS – hydrogen peroxide stress conditions.

### Gingipain activity was decreased in the *PG_0686*-deficient mutant, FLL361.

**FIG 4:**
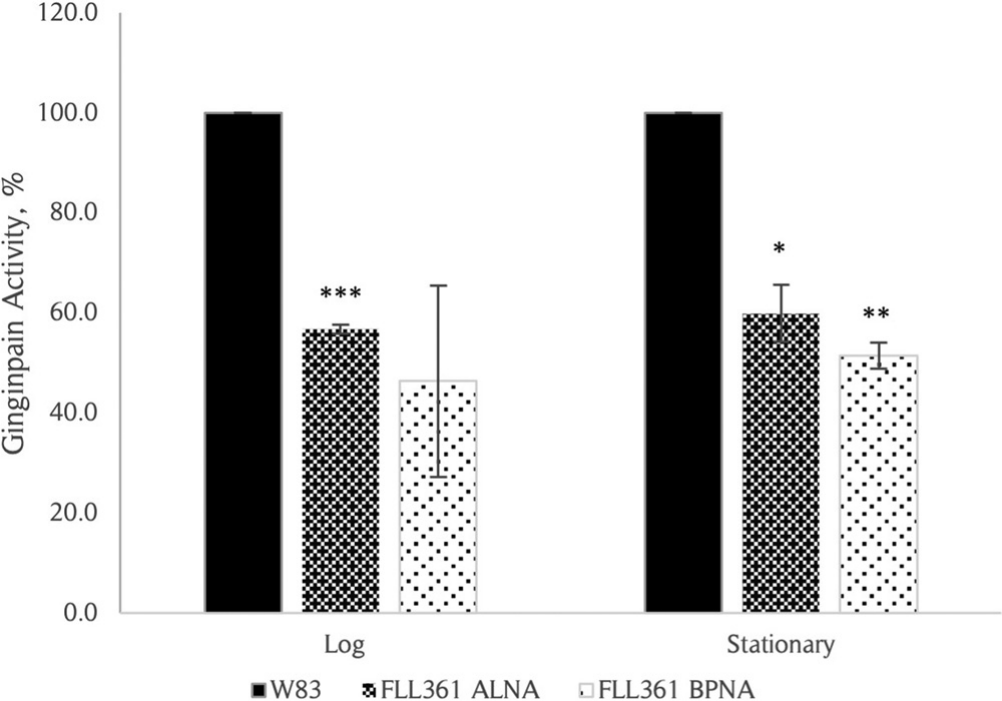
Proteolytic activity in P. gingivalis W83 and FLL361 strains. The whole cell culture Rgp and Kgp activities for W83 and FLL361 strains at log (OD_600_ ~ 0.6) and stationary (OD_600_ ~ 1.4) phases were measured using BPNA and ALNA substrates, respectively. Rgp and Kgp proteolytic activities were reduced in the FLL361 mutant compared to W83 wild type. Results shown represent at least 3 independent experiments, each in triplicate. Error bars indicate standard error of the mean (SEM). (*, *P* < 0.1; **, *P* < 0.05; ***, *P* < 0.005).

Our RNA-seq data showed downregulation of the gingipains, which are major virulence factors, in P. gingivalis FLL361 under both AN and HPS conditions (Table 2). Consistent with the transcriptome data, the Kgp proteolytic activity of FLL361 was reduced to 56.7% and 59.9% in log and stationary phases, respectively; while Rgp activity was decreased to 46.4% and 51.5%, compared to the P. gingivalis W83 wild-type (Fig. 4). The data suggest that during oxidative stress in P. gingivalis, the induction of *PG_0686* is linked to modulating gingipain activity.

### *In silico*, physical, and spectroscopic analysis of the PG_0686 protein indicated a multimeric protein structure.

**FIG 5:**
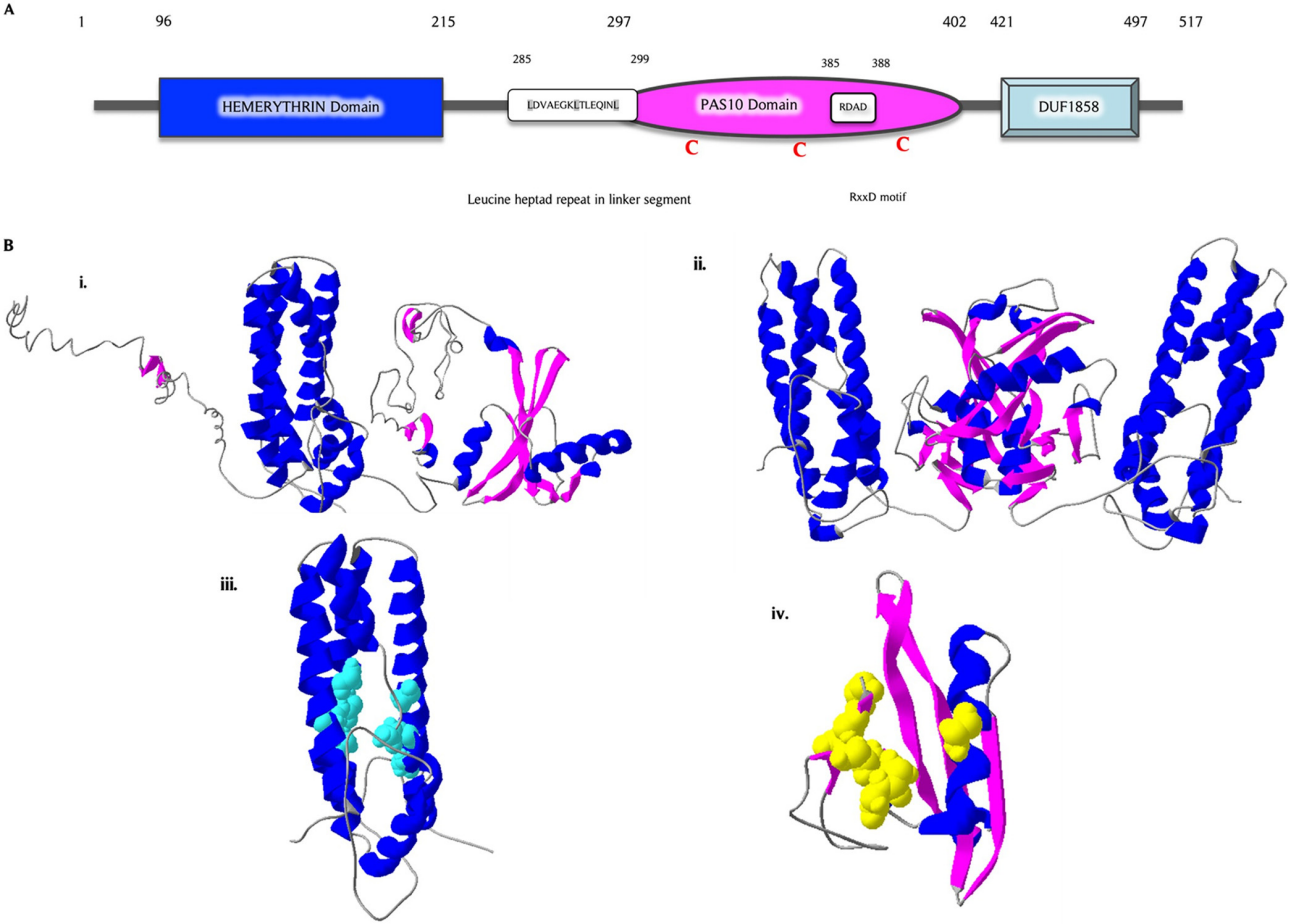
Architecture and *in silico* protein modeling of the hypothetical PG_0686 protein. (A) The domain architecture of the PG_0686 protein, a protein 517 amino acid residues in length, constructed based on BLAST analysis from NCBI and MOTIF Finder databases. PG_0686 does not contain the classic GGD(/E)EF motif found in DGCs. However, the leucine heptad repeat and RxxD inhibitory binding site, typical of DGCs, are observed. (B) *In silico* protein modeling and analysis of PG_0686, (B, panel i.) I-TASSER model of PG_0686, showing protein structure containing helix (blue), coil (gray), and beta sheet (pink) structures. (B, panel ii.) PG_0686 is predicted to be a dimer by SWISS-MODEL. (B, panel iii.) Putative chloro diiron-oxo binding residues (cyan) found in the predicted bacterial hemerythrin (BHr) domain. (B, panel iv.) Putative phosphate binding residues (yellow) found in the predicted PAS 10 domain.

The *PG_0686* gene is 1554 nucleotides in length, and is predicted to encode a 60 kDa hypothetical protein (517 amino acids) of unknown function (https://www.homd.org/genome/genome_description?gid=SEQF1064). This protein is missing any identifiable signal peptide, transmembrane, or DNA-binding domains. Three domains, including hemerythrin (BHr) (96-215), PAS10 (297-402), and DUF-1858 (421-497), were identified in PG_0686 (Fig. 5A). A comparative analysis of the PG_0686 amino acid sequence with other previously studied DGCs (31) showed a characteristic DGC leucine heptad repeat [LDVAEGKLTLEQINL, 285-299] (Fig. S3A) within the coil segment between the hemerythrin and PAS10 domains, and the inhibitory binding RxxD motif [RDAD, 385 to 388] in the PAS10 domain. There were 4 cysteine residues (69, 318, 342, and 401) observed, 3 of which were present in the putative PAS10 domain. Fig. S3B shows the PG_0686 protein sequence with all these features identified and highlighted. Fig. 5B shows the predicted structures and binding partners for the PG_0686 protein. An I-TASSER (32–34) model of PG_0686 predicts a protein with 39% helix and 7% beta-strand. The secondary structure composition of the purified protein was analyzed using circular dichroism spectrometry, which showed 48% helix and 9% beta-strand (data not shown). The SWISS-MODEL (35–37) also predicted a homodimer quaternary structure for PG_0686. The PG_0686 protein was predicted to bind iron and phosphate. I-TASSER predicted a chloro diiron-oxo ligand with binding site residues N105, H141, F142, K145, M165, D169, L202, L205, M206, and E209 in the BHr domain (Fig. S3B). In addition, the PAS10 domain was predicted to bind phosphate at the D312, L316, V317, E338, V339, R340, N341, and E352 residues (Fig. S3B).

A protein-protein BLAST (BLASTP) analysis using the amino acid sequence of the PG_0686 protein shows some similarity with a myriad of proteins, a selected few including DGCs in *Tannerella* sp. (65.6%) and *Candidatus* [*Bacteroides*] *periocalifornicus* (54.7%), as well as PAS domain sensory box proteins from several anaerobic species (Fig. S4A). In a PSI-BLAST analysis of PG_0686, proteins observed to have similar significant features to PG_0686 were hemerythrin HHE cation binding domain proteins from Catonella morbi (50.2%) and Eubacterium brachy (46.9%), as well as DGCs from Fusobacterium nucleatum (48.3%) and Sutterella wadsworthensis (48.2%) (Fig. S4B). PG_0686 also shares features with histidine kinases from *Bacteroidales bacterium* (45.5%), *Geosporobacter ferrireducens* (45.6%), and *Clostridium* sp. (43.5%). PG_0686 shows homology to the bacteriohemerythrin DcrH-Hr and sensory box proteins (ca. 34%) from Desulfovibrio vulgaris (data not shown). A general BLASTP using the hemerythrin domain of PG_0686 was used to generate a phylogenetic tree, which showed the relatedness of PG_0686 to proteins, both cannonical and annotated DGCs, from several bacteria, including anaerobes (data not shown). A template modeling (TM) aligned with selected proteins from this list showed distant relatedness with known DGCs (VC1216, TM = 0.19623, root mean square deviation [RMSD] = 5.31; PleD, TM = 0.21087, RMSD = 7.72), and close relatedness with an annotated diguanylate cyclase protein from *Tannerella* sp. (TM = 0.82718, RMSD = 3.46) (Fig. S5). A TM score was given to each alignment to indicate the strength of the alignment and structural relatedness of the proteins. (0.00 < TM score < 0.30) represented random structural similarity; (0.50 < TM score < 01.00 represented about the same fold). In addition, a RMSD value was assigned to the alignment (as the value approaches zero, the more structurally similar the proteins are). Taken together, these results could indicate that the PG_0686 protein may not belong to the group of cannonical DGCs, and may represent a new class.

Spectrophotometric analysis of the recombinant PG_0686 (rPG_0686) protein indicate 2 characteristic peaks at 410 and 450 nm. The purified rPG_0686 protein was deep brown in color at high concentrations and appeared pale yellow at 50 μM concentration in Tris buffer. Incubation of rPG_0686 with sodium dithionite (Na_2_S_2_O_4_), an oxygen scavenger that reduces O_2_ to the -2 oxidation state, under AN conditions for ca. 3 h resulted in a shift of the characteristic peaks of the rPG_0686 spectra (Fig. S6A). Upon reoxygenation of the protein/dithionite mixture, the characteristic peaks were restored. The data suggest that rPG_0686 can reversibly bind oxygen.

Size exclusion chromatography (SEC) and native-PAGE analyses of the purified rPG_0686 protein suggest 2 native multimeric forms of rPG_0686. Calculations based on SEC analysis showed decameric and pentameric conformations, while native-PAGE analysis showed octameric and tetrameric conformations of the protein. SDS-PAGE and immunoblot analyses of the SEC fractions confirmed the *in silico* predictions, that PG_0686 is a 60 kDa monomer and multimeric under native conditions (Fig. S6B). Mass spectrometry (data not shown) and immunoblot (using polyclonal antibodies raised against rPG_0686) analyses confirmed the identity of the PG_0686 protein.

### Iron was detected in PG_0686.

**FIG 6:**
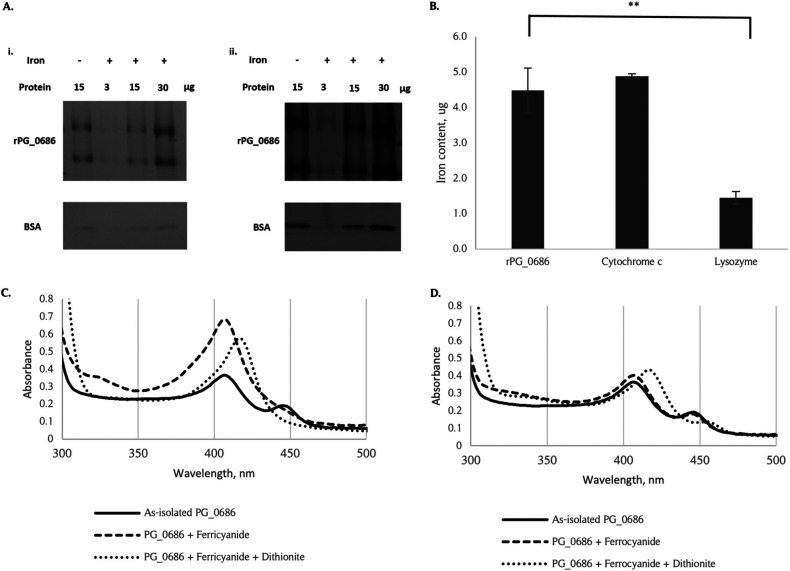
The presence of iron in the rPG_0686 protein. (A) Iron staining of rPG_0686 shows the presence of iron in the protein. Three, 15, or 30 μg of rPG_0686 or BSA was incubated with 0.1 mM Fe(NH_4_)_2_SO_4_•6H_2_O, and then resolved on a native protein gel. (A, panel i.) The gels were then stained with 1 mM 3-(2-pyridyl)-5,6-bis(2-[5furyl sulfonic acid])-1,2,4-triazine (Ferene S), and 15 mM thioglycolic acid in 2% (vol/vol) acetic acid. (A, panel ii.) A duplicate gel was stained with SimplyBlue Safestain to determine equivalent protein loading. rPG_0686 protein not incubated with iron, and BSA were used as controls. (B) EDTA determination of iron content shows the presence of iron in rPG_0686 protein. Cytochrome c was used as the positive control, and lysozyme was used as the negative control. (C) A change in the UV-Vis spectra of rPG_0686, due to the reaction with potassium ferricyanide, indicated a change from oxy-hemerythrin to met-hemerythrin in the protein. Reduction of the mixture with sodium dithionite did not restore the as-isolated spectra, but changed the spectra, likely due to deoxy-hemerythrin in the protein. (D) No change in the UV-Vis spectra of rPG_0686 when incubated with potassium ferrocyanide. Reduction of the mixture with dithionite changed the spectra to the deoxy-hemerythrin profile. (**, *P* < 0.05).

*In silico* analysis of PG_0686 predicts the presence of a non-heme iron group in the BHr domain. Ferene S staining of rPG_0686 in both the presence and absence of incubated Fe(NH_4_)_2_(SO_4_)_2_•6H_2_O shows the presence of iron in the rPG_0686 protein (Fig. 6A). Increasing concentration of rPG_0686 showed an increased iron stain signal. Conversely, increasing concentration of the negative control, bovine serum albumin (BSA), did not show increased iron. The iron content of rPG_0686, determined by EDTA assay (Fig. 6B), also showed the presence of iron in the rPG_0686 protein, similar to the known iron-containing protein cytochrome c. To confirm the oxidation state of the iron present in the protein, rPG_0686 was treated with potassium ferricyanide or potassium ferrocyanide, which will react with Fe^2+^ or Fe^3+^, respectively. The potassium ferricyanide-treated rPG_0686 protein produced a change in the protein spectra (Fig. 6C). Subsequent reduction with sodium dithionite did not revert the spectra to the as-isolated form, but produced a different spectra profile. In contrast, reaction in the presence of potassium ferrocyanide did not change the rPG_0686 protein spectra (Fig. 6D). Since potassium ferricyanide was previously shown to liberate O_2_ from hemerythrin to form met-hemerythrin (38), the change in the protein spectra was most likely due to this reaction. The as-isolated PG_0686 spectra most likely represented oxy-hemerythrin and the sodium dithionite-treated protein spectra represented the deoxy-hemeythrin (39). The met-hemerythrin was not produced by treatment with potassium ferrocyanide. Taken together, the data indicate the presence of iron in the rPG_0686 protein BHr domain.

### The PG_0686 protein showed DGC activity.

**FIG 7:**
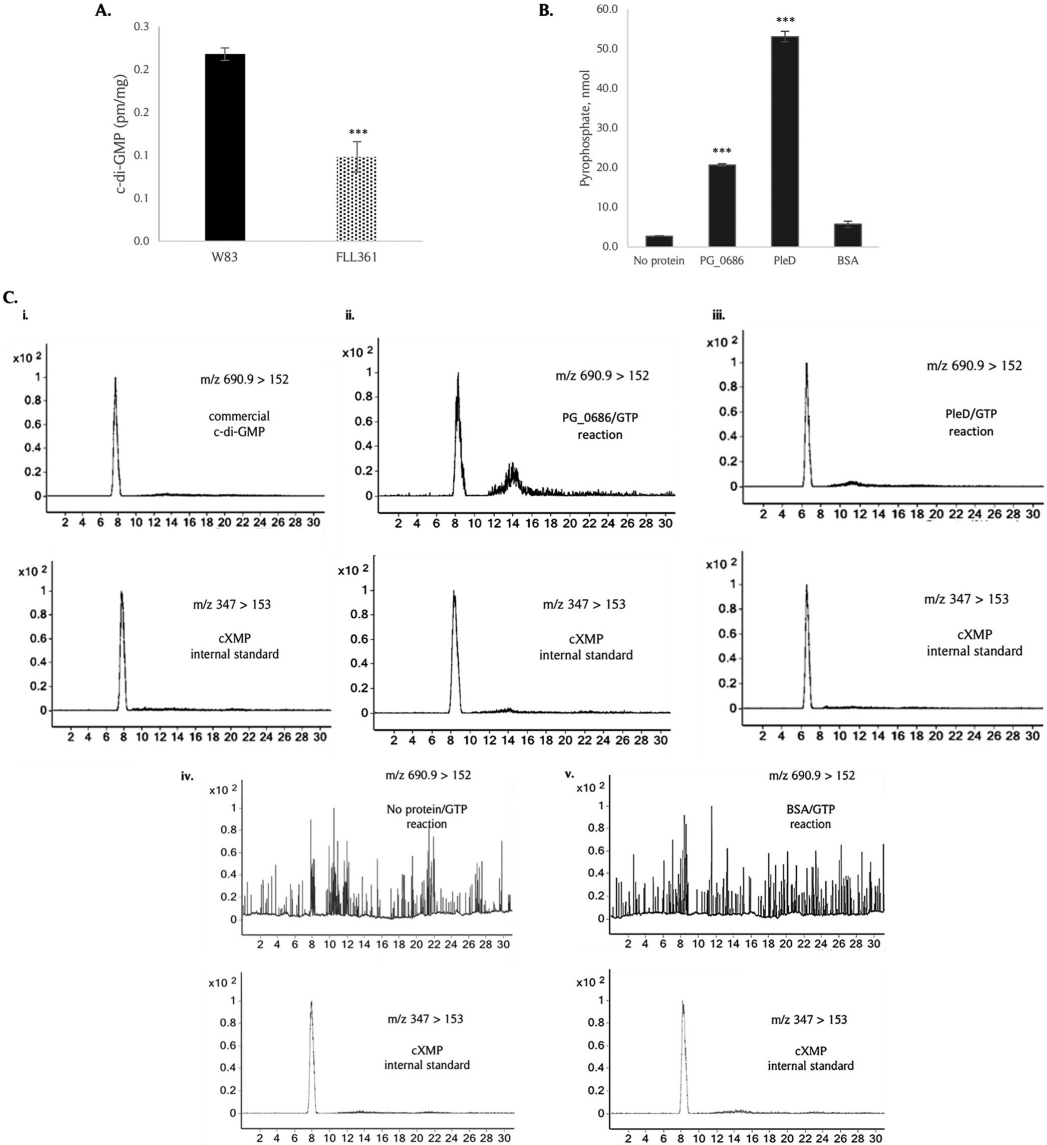
Diguanylate cyclase activity of rPG_0686. (A) C-di-GMP levels in the PG_0686-deficient mutant are reduced compared to the W83 wild type. (B) rPG_0686 shows diguanylate cyclase activity similar to the known diguanylate cyclase, PleD. Bars indicate c-di-GMP or pyrophosphate levels, and error bars indicate SEM. (*, *P* < 0.05; ***, *P* < 0.001). (C) LC-MS/MS analyses of DGC assay reactions indicated that rPG_0686 produced c-di- GMP in the presence of GTP. A total of 3 μg/mL cXMP was added to each sample as an internal standard. Results show MRM transitions *m/z* 347 > 153 and 690.9 > 152 that correspond to cXMP and c-di-GMP in the reaction matrix, respectively. (C, panel i.) Commercial c-di-GMP, (C, panel ii.) PG_0686/GTP reaction, and (C, panel iii.) PleD/GTP reaction showed c-di-GMP peak. No c-di-GMP peak was observed in the (, panel iv.) no protein/GTP reaction and (C, panel v.) BSA/GTP reaction.

To evaluate the effect of PG_0686 on c-di-GMP levels in P. gingivalis, the cell lysate of the FLL361 mutant was compared to that of the wild-type strain. Liquid chromatography combined with tandem mass spectrometry, LC-MS/MS analysis of P. gingivalis the lysates showed a significant reduction (ca. 54%) of intracellular c-di-GMP levels in P. gingivalis FLL361 compared to the W83 wild-type (Fig. 7A). The intracellular c-di-GMP was compared to a commercial standard (Fig. S7A). DGCs catalyze the conversion of 2 GTP molecules to 1 c-di-GMP and 2 inorganic pyrophosphate molecules (2GTP → c-di-GMP + 2PP_i_). We investigated PG_0686 DGC activity by incubating the rPG_0686 protein with GTP, and assaying for c-di-GMP and PP_i_ production. The known DGC from Caulobacter crescentus, PleD (40, 41), was used as a positive control, while BSA was used as the negative control. A sample with no protein was prepared as quality control (QC). We observed the liberation of PP_i_ only in the presence of PG_0686 or PleD (Fig. 7B). In addition, increasing concentrations of GTP or rPG_0686 showed increased release of PP_i_ (Fig. S7B). Thus, our data demonstrated that PP_i_ was formed when rPG_0686 was incubated with GTP. LC-MS/MS analysis was used to evaluate the production of c-di-GMP when rPG_0686 was incubated with GTP (Fig. 7C). Commercially obtained c-di-GMP and cXMP were used as standards (Fig. 7C, panel i.). Our LC-MS/MS data showed that c-di-GMP was formed when rPG_0686 or PleD was incubated with GTP (Fig. 7C, panels ii. and iii.). No characteristic c-di-GMP chromatogram was observed in the no protein QC or BSA control reactions (Fig. 7C, panels iv. and v.). Taken together, these data showed that PG_0686 has DGC activity, which is likely responsible for synthesis of c-di-GMP in P. gingivalis, and may represent a new class of DGCs.

## DISCUSSION

Adaptability to the changing environment of the periodontal pocket is critical for the survival and pathogenesis of P. gingivalis (reviewed in 13). While several earlier reports have documented inducible protective systems in response to environmental stress commonly encountered by bacteria (reviewed in 42), information on the role of previously hypothetical genes in these processes in P. gingivalis is still unclear. Moreover, mechanisms for the involvement of these hypothetical genes is unknown or implied, at best. In this study, we have examined the functional role of one hypothetical protein, PG_0686, in regulating the oxidative stress response, virulence potential, and secondary messenger signaling in P. gingivalis.

Our data has confirmed that the inducible *PG_0686* gene, which encodes for a 60 kDa hypothetical protein, is upregulated in P. gingivalis under conditions of oxidative stress. In addition to HPS, the *PG_0686* gene was also confirmed to be upregulated in response to O_2_- and NO-induced stress. Together with other reports that have shown the upregulation of *PG_0686* in P. gingivalis during its contact with epithelial and invasion of endothelial cells (19, 20), the data suggest a role for this gene in a broad response to environmental stress, possibly mediated by a common mechanism.

The transcriptional profile of the *PG_0686*-defective mutant showed the dysregulation of several genes, including gene clusters/operons and transcriptional regulators. Several transcriptional regulators, including *PG_2212*, *CdhR* (*PG_1237*) and *PG_1181*, were upregulated under AN conditions in the *PG_0686*-defective mutant. Genes (*PG_1551-56*) in the *hmu* operon were the most highly upregulated. The uptake of iron/hemin is important in the growth, survival, and virulence of P. gingivalis (29). While the regulation of the *hmu* heme acquisition/utilization system can be modulated under several environmental conditions (e.g., hemin/iron-limitation, cell density, and biofilm formation), its expression appears to be regulated by a complex, multilayered regulatory network. Part of this regulatory network includes PgFur (43), ECF sigma factor SigH (44), and CdhR (45, 46). CdhR is a transcriptional activator of the *hmu* genes in contrast to other iron-related genes (45, 47). This would also be consistent with the downregulation of *cdhR* in P. gingivalis under microaerophilic conditions, and the concomitant downregulation of the *hmu* operon under those conditions (16). Likewise, based on our data in this study, the dysregulation of the *hmu* genes may likely be directly correlated with the upregulation of the *cdhR* gene in the *PG_0686*-defective mutant. While iron is essential to almost all organisms, tight regulation of its transport/utilization is vital, especially for P. gingivalis in the inflammatory environment of the periodontal pocket. The increased sensitivity of FLL361 to HPS would support a tight regulation of heme acquisition/utilization, and the oxidative/reductive capacity of the bacterial cell, as oxidative damage can be driven by the Fenton reaction. Also upregulated, were genes encoding the alkyl hydroperoxide reductase (*PG_0619* and *PG_0618*), which are also known to be important in oxidative stress resistance in P. gingivalis (48). This contrasts with rubrerythrin (*rbr; PG_0195*), which was the most highly downregulated. Rubrerythrins are non-heme di-iron proteins, belonging to the ferritin-like superfamily, that are involved in oxidative stress defense as peroxide scavengers and intracellular iron sequesters in a wide range of organisms, including P. gingivalis (49, 50). Rubrerythrin may function as part of an alternative to the antioxidant system in the catalase-free P. gingivalis (50, 51). In a previous study (50), a rubrerythrin-defective mutant, *rbr*-, showed increased sensitivity to H_2_O_2_- and O_2_-induced stress. This, it is likely that the increased H_2_O_2_ sensitivity in the FLL361 mutant could be associated with the significant downregulation of the rubrerythrin gene. Our data suggest that PG_0686 is likely a positive regulator of the expression of the rubrerythrin gene in P. gingivalis. Other pathways that may be involved in the oxidative/reductive capacity of P. gingivalis, and modulated by PG_2212 (52) or PG_1181 (18), may also occur in a PG_0686-dependent manner. Taken together, these observations suggest that PG_0686 may have a global, regulatory function.

The ability of microbes to sense and respond to environmental stress, that includes redox changes, occurs primarily through sensitive thiols and metals in a sensor/regulator (53–55). It is likely that the PG_0686 protein may have environmental sensory properties. Our *in silico* structural analysis of PG_0686 has identified a hemerythrin domain and cysteine residues, 3 of which occurs in the putative PAS 10 domain. PAS domains are known to be universal signal sensors for gases (e.g., O_2_) and/or intracellular redox potential (56–58). For example, in the Gram-negative bacterium Shewanella oneidensis MR-1, the sensory box protein, as an O_2_/redox sensor, was shown to be involved in optimization of aerobic growth and transitions to anoxia in the organism (59). Our data show that PG_0686 is an iron-containing protein, a hallmark characteristic of hemerythrins. Hemerythrins, widely distributed in nature, are non-heme, iron-containing, oxygen-binding proteins in which O_2_ binds to a diiron center (58, 60, 61). They feature a conserved motif containing 5 histidine (H…HxxxxE…HxxxH…HxxxxD), and 2 carboxylate residues which, together, coordinate the binding of 2 iron molecules. The characteristic oxo/dicarboxylato-bridged diiron site is in a hydrophobic pocket and surrounded by a four-helix bundle (58, 62). There are some hemerythrin sequences that deviate from the canonical sequence (ca. 18% of 327 bacterial sequences that were analyzed in one study) with one of the ligand binding histidine residues being replaced with one of several other residues (Gln, 34%; A/V/L/I/M/Y, 27%; Asn, 10%; Glu/Asp, 10%) (63). Hemerythrins are also thought to function in binding several compounds, and may have several biological roles. They may bind O_2_ or H_2_O_2_ as a storage, sensory, or detoxification mechanism (49, 64). Similarly, iron, or other metals (such as cadmium), can be bound for storage, detoxification, or function in some other mechanism unrelated to the binding of metals or oxygen (49). In addition, it has been reported that hemerythrins may play a role in intracellular iron homestatsis, and the repair of iron centers in certain bacteria (65, 66). PG_0686 lacks the classic 5 His motif featured in other hemerythrins, and has only one histidine residue predicted to be involved in chloro diiron-oxo binding; as such, it appears to be a part of the group of non-canonical hemerythrin sequences. As of yet, it is unclear whether PG_0686 is able to act as an O_2_ sensor. However, PG_0686 shows ca. 34% homology to the previously studied hemerythrin, DcrH-Hr protein from *D. vulgaris* (67), which is involved in aerotaxis in the organism. The PG_0686 DUF-1858 may also provide sensory properties. This domain is predicted to be found in over 1,000 proteins, and is closely related to a family of redox-disulfide proteins (67). Redox-disulfide proteins include thiol-based redox switches, in which redox-sensitive cysteine residues experience reversible thiol modifications in response to reactive chemical species, and in turn modulate protein function, activity, or localization (68, 69). Cysteine thiol groups may undergo a variety of reversible modifications, which result in the formation of sulfenic acid, inter/intramolecular disulphide, mixed disulphide with glutathione (GSH), *S*-thiolated adducts, sulfenamide, nitrosothiol, or *S*-nitrothiol groups (55, 70, 71). Irreversible modification of the cysteine residue includes the formation of sulfinic and sulfonic acids (55, 72). Reactive oxygen species (ROS), reactive nitrogen species (RNS), and reactive electrophilic species (RES) are all able to interact with redox-active cysteine thiols, making them targets for posttranslational modifications (73). Through these modifications, proteins within sensory systems are able to exploit the complex redox chemistry of cysteine to sense reactive chemical species, and coordinate the expression of appropriate adaptive cellular responses (55). Several proteins have been identified as thiol-based redox switches in both eukaryotes and prokaryotes (55, 68, 74). These proteins can modulate a response to reactive chemical species through varied mechanisms; all geared toward the restoration of a reducing environment (55, 68). It is noteworthy that the DUF-1858 is also present on the hemerythrin DcrH-Hr protein from *D. vulgaris* (67), and could further suggest an O_2_/redox sensor function of PG_0686 in P. gingivalis. The sensory properties of PG_0686 is under further investigation.

Data from this study have confirmed that PG_0686 in P. gingivalis functions as a diguanylate cyclase. This is consistent with a previous study that has indicated the presence of the bacterial second messenger c-di-GMP in P. gingivalis (28). This is in contrast, however, to other reports that have predicted a missing c-di-GMP signaling infrastructure or undetected c-di-GMP in P. gingivalis (23, 27). Our transcriptome data showed the downregulation of *PG_1987*, a GGD(/E)EF protein, in the *PG_0686*-deficient mutant, FLL361. Deletion of the *PG_1987* paralog, *PGN_1932* in P. gingivalis ATCC 33277 strain resulted in the reduction of cellular c-di-GMP (28). Although the widely distributed GGD(/E)EF consensus catalytic domain observed in DGCs is missing in PG_0686, this protein can convert GTP into c-di-GMP and pyrophosphate. Consistent with this observation is the ability of PG_0686 to modulate the cellular level of c-di-GMP in P. gingivalis. The results of our LC-MS/MS analyses confirmed the presence of c-di-GMP in P. gingivalis, and also demonstrated a significant decrease in the intracellular c-di-GMP level in the *PG_0686*-defective mutant compared with the wild-type W83 strain. It should be noted that the PleD protein, used as a positive control in our reactions, was more efficient in the formation of both c-di-GMP and PP_i_. However, the tertiary structure of PG_0686 is very different from PleD (Fig. S5), and, as a result, PG_0686 may not perform well under the reaction conditions used; which were optimized for PleD enzyme activity (40). More investigation is needed to determine the optimum conditions for PG_0686 DGC activity, a process that may be begun by purifying PG_0686 from P. gingivalis. Taken together, our data confirm the involvement of PG_0686 in c-di-GMP synthesis in P. gingivalis. Our results also raise questions about the function of other genes in this process. The low levels of c-di-GMP detected in FLL361 (Fig. 7A), indicates the possibility of a secondary source for its production. PG_1987 is a likely candidate, and further assessment is needed to determine its role in c-di-GMP signaling. In a previous study, in P. gingivalis, PGN_1932 (PG_1987 paralog; 89% identity), the ATCC 33277 strain showed an ability to modulate the intracellular c-di-GMP levels in an isogenic mutant defective in the gene (28). However, the direct involvement of PGN_1932 in the conversion of GTP into c-di-GMP is still unclear. It is noteworthy that PG_1987 was downregulated ca. 4-fold in the *PG_0686*-defective mutant, raising the possibility that it is regulated in a *PG_0686*-dependent manner. Furthermore, we cannot rule out the likelihood that the decrease in c-di-GMP observed in FLL361 could be partly attributed to decreased *PG_1987* expression in P. gingivalis FLL361 (Δ*PG_0686*::*ermF*). Further studies on a *PG_0686-PG_1987* double mutant are necessary to determine the roles and relative significance of each protein in c-di-GMP biosynthesis.

Many DGCs are multiple domain proteins with some of their activities being regulated by environmental signals via sensory domains (21–23). The input signals received via these sensory domains may be necessary for the activation and multimerization of some of these DGCs (31). Hemerythrin is one such domain found in DGCs (31). While hemerythrins are widely distributed in nature (49, 75), the first report of the regulatory function for a bacterial hemerythrin (Bhr) in DGC activity was observed in Vibrio cholerae (61). Vc Bhr-DGC (VC1216) was shown to be significantly more active as a DGC in highly reducing or anaerobic environments, and rapidly convert to a much less active form upon exposure to an aerobic environment. Chimeric hemerythrin proteins often have a GGD(/E)EF domain in the protein as well (63). Our *in silico* analysis shows that the P. gingivalis PG_0686 hemerythrin domain shares a similar architecture to the Vc Bhr-DGC hemerythrin domain (Fig. S5), and while the PG_0686 hemerythrin domain could play a similar role in O_2_/redox sensing, its ability to regulate DGC function in P. gingivalis in currently unknown.

Upon responding to environment signals, c-di-GMP drives bacteria to achieve physiological adaptation through its downstream effectors (both protein and RNA), which will initiate the appropriate cellular action (76, 77). Both positive (activation) and negative (repression) regulation via this second messenger may occur at the transcriptional, translational, and post-translational level, and through an increasing number of c-di-GMP-binding riboswitches and proteins (25). Transcription factors can act as receptors of the second messenger (78–80). Moreover, there is some evidence that the c-di-GMP signal can modulate oxidative stress resistance in bacteria via an integration of functionally divergent transcription factors into a regulation pathway for antioxidant defense (80). The dysregulated oxidation/reduction capacity of P. gingivalis FLL361, in both a positive and negative manner, could be consistent with or implicate a PG_0686-dependent second messenger regulatory system in P. gingivalis. In the absence of *PG_0686*, increased gene expression under non-inducing conditions could indicate the release of a repressor function. For example, the upregulation of the *PG_1551-PG_1556* (*hmu*) gene cluster and alkyl hydroperoxide reductase genes could suggest that a transcriptional repressor element might be involved in their observed dysregulation. Because CdhR is a novel transcriptional activator for the *hmu* operon (45), which can be differentially expressed under specific environmental conditions (16, 81) and is part of a complex regulatory network (46), it could likely be a c-di-GMP effector as part of a mechanism of its regulation. In the absence of *PG_0686*, decreased gene expression would indicate positive regulation and the possible requirement for the binding of a putative factor (most likely a transcription factor) to the promoter of the affected genes. Although several hypothetical genes were downregulated under normal conditions in the *PG_0686*-defective mutant, there were no discernible transcriptional regulators. There are currently no known transcriptional regulators for the gingipain and rubrerythrin genes, which were also downregulated. We cannot rule out, however, that some of the modulated transcriptional factors might also be involved in the transcriptional activity of these genes. Upon responding to the c-di-GMP signal, transcription factors can positively or negatively affect gene expression, as c-di-GMP can directly stimulate or inhibit DNA-binding activities. For example, in Mycobacterium smegmatis, low concentrations of c-di-GMP stimulate the DNA-binding activity of HpoR, whereas high concentrations of the signal molecule inhibit the activity in its antioxidant defense system (80). It is unclear if there is a similar role for some of the modulated transcriptional regulators in the *PG_0686*-defective mutant. This is under further investigation in the laboratory.

As a second messenger and a global signaling molecule, c-di-GMP is part of a signaling system that is important, and well-conserved in all major bacterial phyla (21–24). Based on the classical conserved motifs of the vital components (including PilZ proteins, GGD(/E)EF, and/or EAL domain-containing proteins, and riboswitches) for this system, the P. gingivalis genome is missing the classical genes involved in c-di-GMP signaling (23). However, the P. gingivalis genome carries genes that encode for products that could be part of a 3′,5′-cyclic dimeric AMP (c-di-AMP) signal transduction system as well, which constitutes an atypical c-di-AMP signaling system in the microbe (23, 27). Our study has provided direct evidence that the P. gingivalis PG_0686 protein can catalyze c-di-GMP synthesis from GTP, and plays a role in modulating the intracellular levels of c-di-GMP in this bacterium (Fig. 7). The impact of this c-di-GMP intracellular modulation appears to be the dysregulation of the environmental/oxidative stress resistance and virulence capacity of P. gingivalis. The novel PG_0686 P. gingivalis protein, with its putative sensor capabilities, has homologs in other anaerobic bacterial species that are also missing the classical c-di-GMP signaling system (22, 23). In addition, despite PG_0686 not having the classic GGD(/E)EF motif, bioinformatics shows other features of DGCs, such as the leucine heptad repeat and RxxD inhibitor domain. One of the homologs of PG_0686 is present in *Tannerella* sp. (WP069175758.1, 64.4% identity). It has a high TM score of 0.8 with PG_0686 (RMSD = 3.46), and also features the leucine heptad repeat and RxxD inhibitor domain, while lacking the GGD(/E)EF motif. Alignment with PG_0686 paralogs in other anaerobic species (data not shown) also shows these DGC features along with several conserved residues across species, which suggest that PG_0686 represents a novel class of DGCs and the corresponding pathway. A phosphodiesterase that may couple with PG_0686 in c-di-GMP metabolism has yet to be identified. However, the genome of P. gingivalis W83 carries 2 genes previously annotated as phosphodiesterases, but their roles in c-di-GMP metabolism in P. gingivalis are unknown. Taken together, our results suggest a global regulatory property for PG_0686 that may be part of an unconventional second messenger signaling system that can coordinately regulate pathways vital for protection against environmental stress, and is significant in the pathogenicity of the P. gingivalis and likely other anaerobes. It is of interest to determine the structural and functional properties of PG_0686, and to identify and confirm the relevant effectors and downstream modules. How PG_0686-dependent c-di-GMP signaling integrates functionally divergent transcriptional regulators, or other effectors into a regulatory pathway for oxidative stress defense and virulence in P. gingivalis needs further study. Characterization of a global regulatory network in P. gingivalis is prerequisite to the development of novel therapeutic interventions to aid in the control and prevention of diseases associated with this keystone and other periodontal pathogens.

## MATERIALS AND METHODS

### Bioinformatics analysis.

DNA and amino acid sequences of *PG_0686 gene* from P. gingivalis W83 were retrieved from the NCBI database, Oralgen (https://www.homd.org/genome/genome_description?gid=SEQF1064), aligned using Bioedit (https://bioedit.software.informer.com), and were analyzed using MEGA version 7.0 (82). The amino acid sequences were analyzed using Clustal Omega version 2.0 (http://www.ebi.ac.uk/) (83), and the secondary structure prediction and modeling of the protein was performed using the I-TASSER (32) and SWISS-MODEL (35). The models were validated and manipulated using the Swiss-PbdViewer program (37). The domain architecture was predicted using MOTIF Search (https://www.genome.jp/tools/motif/) (84). Metabolic pathway analysis was carried out using the KEGG (www.genome.jp/kegg/) (85), based on the information from the online database ExPASy (86, 87).

### Bacterial strains, plasmids, and culture conditions.

Strains and plasmids used in this study are listed in Table S2, and were cultured as described previously (88). P. gingivalis strains were cultured at 37° C in Brain Heart Infusion (BHI) broth (Difco Laboratories), and supplemented with yeast extract (5 mg/mL), DL-cysteine (1 mg/mL) (Sigma), Vitamin K_3_ (0.5 μg/mL), and hemin (5 μg/mL) (Sigma). All P. gingivalis strains were cultured under anaerobic conditions (10% H_2_, 10% CO_2_, 80% N_2_) in an anaerobic chamber (Coy Manufacturing). E. coli strains were cultured aerobically at 37° C in LB (Difco Laboratories) broth. When using solid media, BHI was supplemented with 20 g/L agar (Fisher BioReagents) and 5% sheep’s blood (Hemostat Laboratories), where indicated. Growth rates for P. gingivalis strains were determined spectrophotometrically by measuring optical density at 600 nm (OD_600_). Erythromycin (10 μg/mL), tetracycline (0.3 μg/mL), and/or ampicillin (100 μg/mL) were added to the media when necessary.

### Construction of the *PG_0686*-deficient mutant, FLL361.

Inactivation of the P. gingivalis *PG_0686* gene was performed as described by Dou et al. (89). The primers used are listed in Table S3. The replacement of *PG_0686* by *ermF* in the erythromycin-resistant mutants was confirmed by colony PCR and DNA sequencing (Retrogen Inc.). The resulting *PG_0686* mutant was designated as FLL361.

### Complementation of *PG_0686*-deficient mutant, FLL361.

A DNA fragment containing the *PG_0686* open reading frame (ORF) was amplified from chromosomal DNA of P. gingivalis W83, using primer sets *PG_0686* Fwd and *PG_0686* Rvs (Table S3). Promoter prediction software was used to determine possible *PG_0686* promoter regions. A SalI restriction site was designed at the 5′ of both primers to facilitate the subcloning of the PCR fragment. The pT-COW plasmid was SalI-digested and ligated to the PCR fragment, and then used to transform E. coli DH5α. The purified recombined plasmid, designated pFLL361a, was used to transform P. gingivalis FLL361 (Δ*PG_0686*::*ermF*) by electroporation. Aliquots were plated on BHI agar supplemented with 10 μg/mL erythromycin and 0.3 μg/mL tetracycline. The complemented strain was designated FLL361’C.

### Confirming *PG_0686* gene induction.

To validate the previously published microarray gene expression data (15), which indicates induction of *PG_0686* gene under HPS, total RNA from P. gingivalis W83 cells treated with 0.25 mM H_2_O_2_ for 15 min were subjected to RNA-sequencing, RT-PCR, and real time RT-PCR. The cultures were grown to ca. OD_600_ 0.6, and then split in half. One half was left untreated while the other was treated with 0.25 mM H_2_O_2_ for 15 min. After this, total RNA was immediately extracted from both the untreated and treated portions. Total RNA from each sample was then subjected to RT-PCR analysis using primers for the *PG_0686* and 16S rRNA genes (Table S3).

### qRT-PCRs.

Amplification of selected genes was performed using the Transcriptor High Fidelity cDNA Synthesis Kit (Roche) and the QuantiTect SYBR Green PCR Kit, and real-time fluorescence signal was detected by using the Cepheid Smart Cycler II Real Time PCR apparatus (Cepheid). qRT-PCRs were performed as follows: 95°C for 15 min, followed by 40 cycles of 94°C for 15 s, 54°C for 30 s, and 72°C for 30 s. Each measurement was performed in triplicate for each gene. The 16S rRNA gene was used as an internal control to normalize variations due to differences in reverse transcription efficiency. Where applicable, the comparative cycle threshold (C_T_) method, as described by Livak and Schmittgen (90), was used to quantify fold change, using the formula 2^-ΔΔCT^.

### Hydrogen peroxide sensitivity and survival assays.

Sensitivity of P. gingivalis strains to H_2_O_2_ was tested as previously reported (88). The OD_600_ was measured at specific intervals over the 24-h period. Plates were incubated anaerobically for 5 to 7 days at 37°C. Cell cultures without H_2_O_2_ were used as controls. Colonies were counted, and the percentage of survival was determined. In a similar way, assays for sensitivity of P. gingivalis strains to O_2_/Air for 1 h or NO (DEA NONOate) for 15 min were performed. Experiments were done in triplicate, and at least 2 independent experiments were conducted.

### Proteolytic activity assays.

The activities of Rgp and Kgp gingipain proteases were determined by using a method previously reported (52). Briefly, arginine gingipain activity was measured with 1 mM BAPNA (*N*α-benzoyl-DL-arginine-*p*-nitroaniline) as the substrate in an activated protease buffer (0.2 M Tris-HCl, 0.1 M NaCl, 5 mM CaCl_2_, 10 mM l-cysteine, pH 7.6). Lysine gingipain activity was measured with ALNA (Ac-Lys-*p*-nitroanilide HCl) as the substrate. After incubation of the substrate and culture, the reaction was stopped by the addition of 50 μL of glacial acetic acid. The OD_405_ was then measured against a blank sample containing no protease.

### RNA-seq library construction and sequencing.

The library was generated using RiboMinus Transcriptome isolation kit (Life Technologies) and NEXTflex RNA-Seq Kit (Bioo Scientific), following the vendors’ protocols as described previously (18). Briefly, 2 μg total RNA (20 μL) was hybridized with 4 μL of RiboMinus probe in 100 μL hybridization buffer at 37°C for 5 min, which was then cooled on ice for at least 30 s. The sample was transferred into an Eppendorf tube containing RiboMinus Beads resuspended in 100 μL hybridization buffer, which was incubated at 37°C for 15 min to allow beads to bind with probes. The tube was placed on a magnetic separator for 1 min to pellet the rRNA-probe complex. The supernatant containing RiboMinus RNA was transferred into a new tube. The RiboMinus RNA was concentrated by ethanol precipitation, and resuspended in 14 μL nuclease free water. Five μL of NEXTflex RNA Fragmentation buffer (Bioo Scientific) was mixed with the 14 μL RiboMinus RNA. The mixture was incubated at 95°C for 10 min, and then chilled on ice immediately. The first and second strand of cDNAs were synthesized, following the Bioo Scientific protocol, and the NEXTflex RNA-Seq barcode was ligated to double strand cDNA after the end repair and adenylation for multiplexing. The library was amplified by PCR. The quality of each RNA-seq library was checked using Agilent Bioanaylzer and DNA 1000 Nano chip, and the library was quantified using Qubit (Life Technologies). The libraries (20 samples) were loaded into a single lane of Illumina V3 flow cell for cluster generation, and the sequencing was done on an Illumina NextSeq550 with 100 BP × 2, paired-ends, and 10M reads.

### RNA-seq data analysis.

The quality of reads was checked using FastQC (http://www.bioinformatics.babraham.ac.uk/projects/fastqc/). Raw reads were filtered by Trimmomatic-0.22 (http://www.usadellab.org/cms/index.php?page=trimmomatic), including removing: (i) reads with sequencing adapters; (ii) the 5′-end 5 bases of each read; (iii) reads with more than 20 bases of `N′ nucleotides; (iv) reads with low quality; and (v) reads with length less than 36 bases after trimmed. Trimmed reads were mapped to P. gingivalis W83 genome (NCBI accession ID: NC_002950) using Bowtie2 (https://bowtie-bio.sourceforge.net/bowtie2/index.shtml). The mapped reads were then processed using Cufflinks (http://cufflinks.cbcb.umd.edu/) for transcript assembly. Read counts for each annotated gene in the P. gingivalis W83 genome assembly version ASM758v1 were calculated, normalized as reads per million mapped reads (RPM), and then transformed into log_2_ scale. To avoid infinite values, 1 count was added to each gene before log_2_ transformation. Differentially expressed genes (DEGs) were determined with a *P*-value cutoff of ≤0.05 and a fold change (FC) ≥ 2 (both upregulated and downregulated), compared with wide type W83. The Gene Ontology (GO) functional clustering and pathway analyses were carried out using NCBI DAVID Bioinformatics Tool (https://david.ncifcrf.gov/home.jsp) and ShinyGO (http://bioinformatics.sdstate.edu/go/).

### Overexpression and purification of rPG_0686 and PleD proteins.

The ORF of the *PG_0686* gene was PCR-amplified (Table S3) and cloned in-frame into the pEXP5-NT/TOPO expression vector (Table S2) (Invitrogen). The ligated product was used to transform E. coli Top10, and then plated on LB agar (Sigma-Aldrich) supplemented with 100 μg/mL ampicillin. A recombinant plasmid was isolated and selected for expression, after the presence and orientation of the insert was confirmed by colony PCR and sequencing (Retrogen Inc.). The recombinant plasmid, pEXP5-NT-0686, was then transformed into competent BL21 Star (DE3) pLysS for overexpression of the recombinant PG_0686 (rPG_0686). Overexpression of pEXP5-NT-0686 was induced by 1 mM isopropyl-β-d-thiogalactopyranoside (IPTG) at 16°C. The expression of the rPG_0686 was confirmed by visualization on SDS-PAGE of supernatant, cell lysate, and cellular debris fractions. PleD, a known diguanylate cyclase from C. crescentus, was overexpressed similarly from E. coli (pRP89: pET11-PleD*His6, strain with plasmid gifted by Kazmierczak lab, Yale University).

The rPG_0686 or PleD containing a His6 tag at the N terminus was purified from E. Coli BL21 cell lysate by using nickel-nitrilotriacetic acid (Ni-NTA) resin (Qiagen), according to the manufacturer’s guidelines. Briefly, rPG_0686-containing cell lysate was passed through a column with Ni-NTA resin in binding/washing buffer (50 mM Na-phosphate [pH 8.0], 300 mM NaCl, 0.01% Tween 20) at room temperature, and then washed four times with binding/washing buffer. Proteins binding to the NTA beads were eluted by using elution buffer (500 mM imidazole, 50 mM Na-phosphate [pH 8.0], 300 mM NaCl, 0.01% Tween 20). Eluates were then analyzed by SDS-PAGE and immunoblot analysis. Fractions containing purified rPG_0686 protein were concentrated by buffer exchange dialysis against 10 mM Tris-HCl.

### SEC and immunoblot analyses of purified rPG_0686.

To observe the size of PG_0686 under native conditions, SEC was performed using the ÄKTAdesign ÄKTA FPLC system (Amersham Biosciences) equipped with Superdex 200 10/300 GL columns (GE Healthcare), as previously described (91). Briefly, 100 μL of a 0.2 mg/mL rPG_0686 solution was injected, and a degassed and filtered 0.15 M ammonium bicarbonate solution was used as the mobile phase at a flow rate of 0.1 mL/min. Upon exiting the column, the eluate was directed to a UV absorption detector (operated at 280 nm), and the fractions were collected in 1 mL aliquots. The Bio-Rad 1511901 protein standard (Bio-Rad) was used to calculate sample molecular weights. The fractions were analyzed by Native- and SDS-PAGE gels and immunoblots.

SDS-PAGE was performed as previously described (92). The separated proteins were then transferred to Bio-Trace nitrocellulose membranes (Pall Corporation), and processed at 15 V for 35 min with a SemiDry Trans-blot apparatus (Bio-Rad). The blots were probed with primary antibodies against the rPG_0686 protein (rabbit) (Invitrogen, Inc.), and the secondary goat, anti-rabbit antibodies were horseradish peroxidase conjugated (Zymed Laboratories). Immunoreactive proteins were detected by the procedure described in the Western Lightning Chemiluminescence Reagent Plus kit (Perkin-Elmer Life Sciences).

### Iron detection assays.

Iron staining of rPG_0686 was done as previously described (88). Briefly, 3 μg, 15 μg, or 30 μg of rPG_0686 or BSA were incubated with 0.1 mM Fe(NH_4_)_2_(SO_4_)_2_•6H_2_O for 30 min. The proteins were then resolved by PAGE on 3 to 12% native polyacrylamide protein gel. Protein not incubated with iron was included as a control. The gels were then stained with 1 mM 3-(2-pyridyl)-5,6-bis(2-[5furyl sulfonic acid])-1,2,4-triazine (Ferene S; Sigma-Aldrich) and 15 mM thioglycolic acid in 2% (vol/vol) acetic acid. The gel was subsequently stained with SimplyBlue Safestain (Invitrogen,) to determine equivalent protein loading.

EDTA determination of iron content was modified from previously described (93). A standard curve was created using Fe(NH_4_)_2_(SO_4_)_2_•6H_2_O. Briefly, 150 μL of 2 N HCl was added to 500 μL protein sample. A total of 20 μL thioglycolic acid was then added, and the mixture incubated at room temperature for 30 min. Ice-cold trichloroacetate was then added to a final concentration of 5%, and the mixture was added to a centricon filter and then centrifuged at 3000 rpm for 45 min. The pH of the reaction was then adjusted to 3.6 to 5.6 by adding saturated sodium acetate. The filtrate was then aliquoted, added to 1 mM EDTA (100 μg), and then incubated at room temperature for 30 min. The absorbance was read at 254 nm.

Uv-vis analysis of rPG_0686 Prussian blue reaction was performed as follows: Briefly, 4 μL of a 10 mM potassium ferricyanide (K_3_[Fe(CN)_6_]) or potassium ferrocyanide (K_4_Fe(CN)_6_)(•3H_2_O) was added to 500 μL of 50 μM rPG_0686 solution that was reduced overnight in an anaerobic chamber. The mixture was incubated for 30 min in the chamber, and the spectra was read from 200 nm to 700 nm in a gas-tight cuvette, using the METTLER TOLEDO UV5 spectrophotometer. Reduction of the Prussian blue reaction was performed by adding 25 μL of 50 mM sodium dithionite, and incubating 3 h to overnight in an anaerobic chamber.

### Diguanylate cyclase assay.

The diguanylate cyclase assay was modified from a procedure previously described (40). The standard reaction mixtures contained 250 mM NaCl, 10 mM MgCl_2_, 10 mM NaF, 1 mM BeCl_2_, and 50 μM purified PleD, rPG_0686, or BSA protein in a 200 μL volume. A total of 50 μM GTP (Sigma-Aldrich) was added to make a 250 μL volume. A sample with no protein was prepared as QC. The samples were incubated overnight to 48 h, then the reaction was stopped with 95°C for 5 min and placed on ice for 15 min. The samples were centrifuged at 14,000 g for 30 min to collect the precipitated protein, and the supernatant was removed to a fresh tube for pyrophosphatase or LC-MS/MS analysis.

### Pyrophosphatase analysis of DGC assay samples.

The pyrophosphatase assay was performed using the EnzChek Pyrophosphatase assay kit (Invitrogen), following the manufacturer’s instructions. Briefly, 200 μL of the DGC reaction was used in the assay, and a pyrophosphate standard curve was constructed using the pyrophosphate standards provided in the kit. The DGC assay reaction matrix, containing NaCl, MgCl_2_, NaF, and BeCl_2_ as in the above assay, was used to make up the volume to 350 μL, instead of water. The reaction was incubated at room temperature for 1 h, and then read at 360 nm, in triplicate, using the xMark Microplate Spectrophotometer (Bio-Rad). A modified Fiske–Subbarow assay (94) was also used to evaluate the PP_i_ content in reactions containing increasing concentrations of GTP or rPG_0686.

### LC-MS/MS chemicals.

Aqueous buffer filtration was performed using sterile Whatman filter paper (Sigma-Aldrich). Solvents (ethanol, methanol, phenol, chloroform, and isoamyl alcohol) used for extraction and liquid chromatography (LC) analysis were purchased from Fisher Scientific. Further, 3′,5′-cyclic diguanylic acid (purities > 95%; c-di-GMP) sodium salt (Sigma-Aldrich) was used as a standard. Xanthosine 3′,5′-cyclic monophosphate (cXMP) (Sigma-Aldrich; purity > 95%) was used as an internal standard for calibration of cellular (95) and enzyme-generated c-di-GMP. The concentration of aqueous stock solutions of c-di-GMP and cXMP were 1 mM and 1.77 mM, respectively.

### LC-MS/MS analysis of DGC assay samples.

Quantification of intracellular c-di-GMP levels in P. gingivalis FLL361 and W83 strains was performed as previously described (96), with a change from negative to positive electronspray ionization (ESI). Briefly, overnight cultures were diluted to ca. OD_600_ 0.2, and grown to mid-log phase (ca. OD_600_ 0.6). The cells were pelleted by centrifugation, and then resuspended in extraction buffer (40% methanol and 40% acetonitrile in 0.1N formic acid). The resuspended mixtures were incubated for 30 min at −20°C, and the insoluble material was removed by centrifugation at 4°C. The supernatants were neutralized by the addition of 4 μL of 15% NH4 HCO3 per 100 μL of sample. Each sample was analyzed using LC-MS/MS.

The DGC protein assays were analyzed by LC-MS/MS as previously described (95, 97), with some modifications. Chromatographic separation was performed by using a liquid chromatography system (Agilent Technologies), whereby sample aliquots of 20 μL each were injected via autosampler onto an InertSustain C18 HP 3 μm 2.1 mm × 150 mm column (GL Sciences Inc.) at 30°C. All analyses were conducted in gradient mode with original starting conditions of 97% 10 mM ammonium acetate in water and 3% 10 mM ammonium acetate in methanol, that were changed to 25% 10 mM ammonium acetate in water and 75% 10 mM ammonium acetate in methanol through 30 min at a flow rate of 0.1 mL/min. Re-equilibration of the column between analyses was achieved by a gradient from 25% 10 mM ammonium acetate in water to 97% for 30 min for a total running time per analysis of 60 min.

Analyte detection was performed on a triple quadrupole mass spectrometer (Agilent Technologies) equipped with an ESI source that was inline with the LC. Analyses were conducted in positive ionization mode using nitrogen gas as the nebulizing gas. The ion source temperature was 130°C, desolvation temperature at 350°C, and cone voltage at 35 V. Nitrogen gas was also used as the desolvation gas (600 l/h). The MS was operated in multiple reaction monitoring (MRM) mode using dwell times of 40 ms, and were monitored for c-di-GMP: 690.9 > 152 and for cXMP: 347 > 153, and collision energies were 61 eV and 29 eV, respectively (95). The Masshunter Quantitative and Qualitative Analysis software were used to analyze the data, and quantify c-di-GMP concentrations in the samples.

### Statistical analysis.

All experiments were performed in triplicate for each condition, and repeated at least three times, unless otherwise stated. Error bars represent the standard deviation from the mean. Raw data from sensitivity and survival experiments, RNA-seq, and proteolytic activity assays were processed in MS Excel, and statistical analyses were performed to determine *P* values by using a paired, two-tailed *t* test; while for the pyrophosphate assay, an unpaired assuming unequal variances, two-tailed *t* test was used.

### Data availability.

RNA-seq data were submitted to the Gene Expression Omnibus database (http://www.ncbi.nlm.nih.gov/geo, access number: GSE212414).
