# Supplementary material for: The Oxidative Stress-Induced Hypothetical Protein PG_0686 in Porphyromonas gingivalis W83 Is a Novel Diguanylate Cyclase
Source: Microbiol Spectr. 2023 Jan 31;11(2):e04411-22. doi: 10.1128/spectrum.04411-22 (PMC10101095; doi:10.1128/spectrum.04411-22)
Supplement: Supplemental file 1 — Supplemental material. Download spectrum.04411-22-s0001.pdf, PDF file, 2.0 MB [file spectrum.04411-22-s0001.pdf]

The oxidative stress-induced PG_0686 hypothetical protein in *Porphyromonas gingivalis* W83 is a novel diguanylate cyclase

SUPPLEMENTAL FIGURES

A

W83	+		+		+		+					
FLL361		+		+		+		+				
F_1R_3	+	+							+			
PG0686			+	+						+		
Erm					+	+					+	
16S rRNA							+	+				+

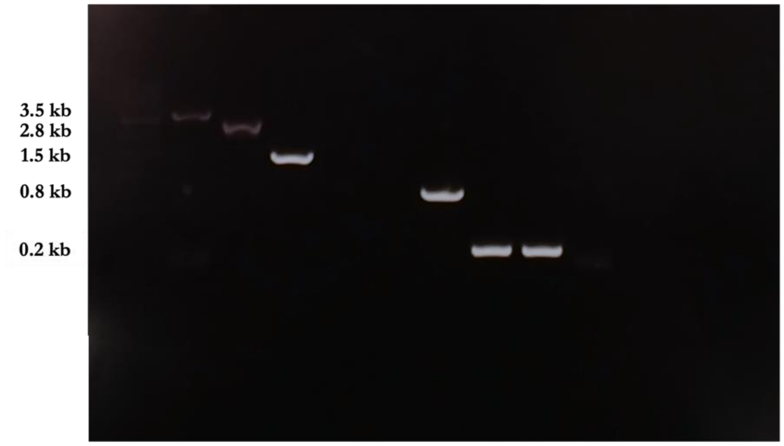

B

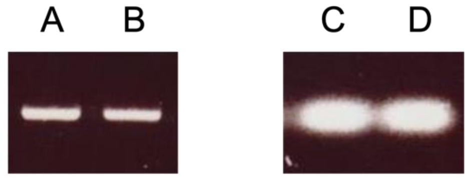

SUPPLEMENTAL FIGURE 1 The creation of the FLL361 mutant. (A) PCR, amplification from chromosomal DNA, confirming the creation of the FLL361 mutant. The *PG_0686* gene was present in the *P. gingivalis* W83 wild-type but absent, and replaced by *ermF*, in the FLL361 mutant. **(B)** RT-PCR shows that PG_*0686* is present but not Induced under H_2O_2 stress in the complemented strain FLL361C'. A – cDNA from Untreated C361' culture; B – cDNA from Treated C361' culture; C – 16S r cDNA control from Untreated C361' culture; D – 16S r cDNA control from Treated C361' culture.

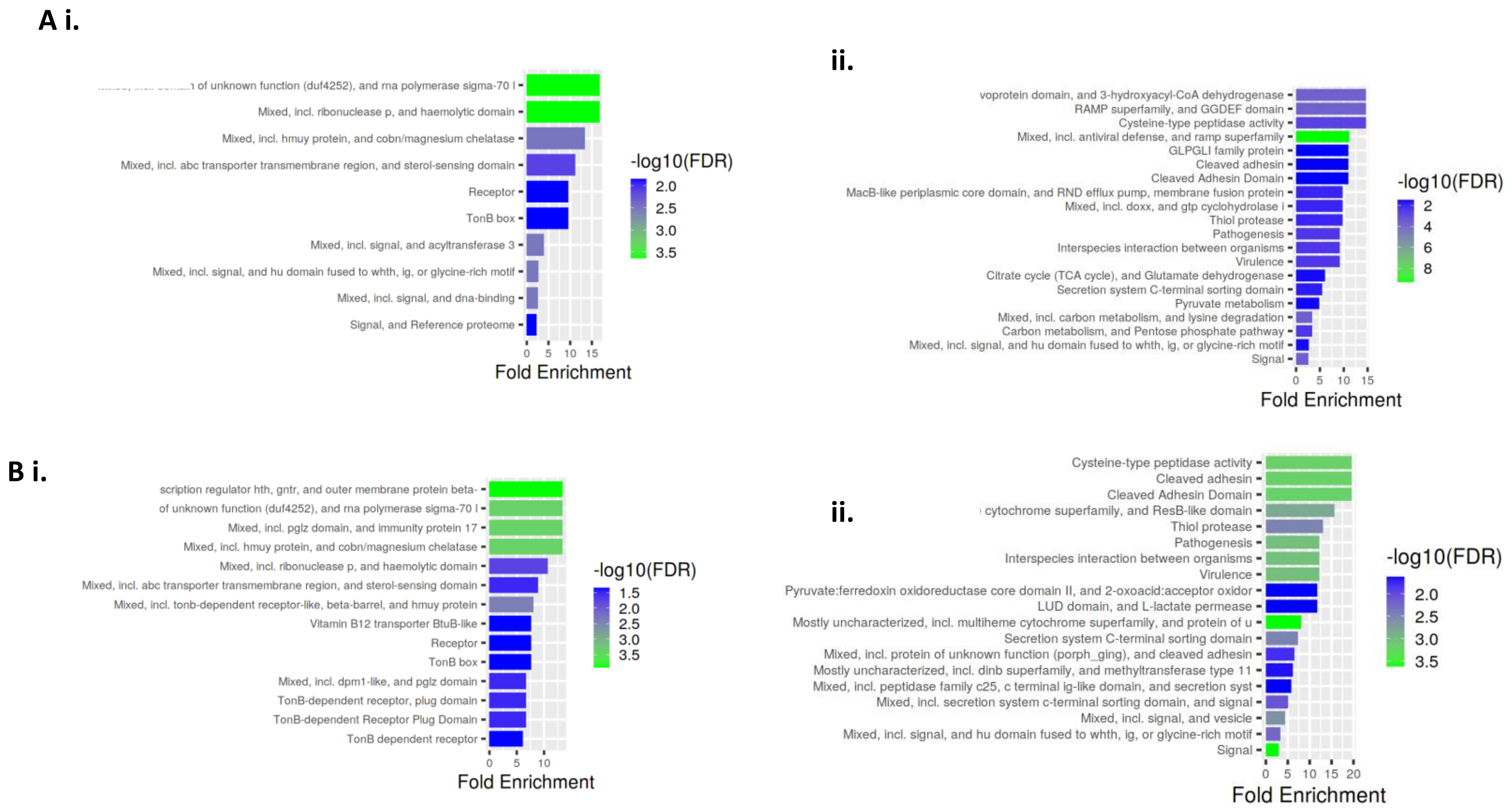

SUPPLEMENTAL FIGURE 2 Gene enrichment from RNA-seq data. (A) The top enriched pathways for differentially upregulated (i) and downregulated (ii) genes in *P. gingivalis* FLL361 under normal anaerobic conditions. **(B)** The top enriched pathways for differentially upregulated (i) and downregulated (ii) genes in *P. gingivalis* FLL361 under HPS conditions. Enrichment plots were generated by ShinyGO 0.76 (Ge, et al., 2020).

A

```
PG0686   KEAHGQSFMAELGALLAKHGMGTGGQDDKAILDVAEGKLTLEQINLLFRHLPVDISFVDENELVCFYT

pleD     PQELSARVKTQIQRKRCTDYLRNNLDHSLELAVTDQLTGLHNRRYMTGQLDS

WspR     ELVARIRYHSRSYMTLLQRDAAYRALRVSQQQLLDTNLVLQRLMNSDGLTGLSNRRHFDEYLELE

Rrp1     SRIIDARVKTHLELKRLRDYFKSLSRIDGLTQIPNRRFFMDKFSKS

3ign     QNLTISPITAPNGEVEKVCLLVYDVTEFASSKRALERANEQLAKLSMTDRLTGLLNRGTWENLVDA

DgcZ     CGRELMLAIVENHWQDAHFDAFQEGLLSFTAALTDYKIYLLTIRSNMDVLTGLPGRRVLDESFDH

VC1216   FDQVSERNRELVKLNQSLEEKVIERTQQLYQVNRQLEALSMTDSLTGLPNRRKAMRQL
```

B

```
1     MQVIKTNETFDSLDKSKLERMLDIKEAHREGHLTLEEAKERMKKEVGSISPEEFAAAEQLFKERDQDECQ   70

71    NEDVRTMLQLFEGLINPIRPDLPFGHPIDAYLRENDKAKELLDQADALLERTFIPNPWIELMETLMGYKL   140

141   HFARKQNQLYSTLEQKGFDRPSTTMWTYDDHIRDEMNKAMSLLREKDYDSFPAAYKEMAIVLRDLMEKEE   210

211   LILYPISLKLISDKEFEEMKHGDREIGFFLIDMPELDAPAKQSKEAHGQSFMAELGALLAKHGMGTGGQD   280

281   DKAILDVAEGKLTLEQINLLFRHLPVDISFVDENELVCFYTDTKHRVFPRSKGVIGREVRNCHPPKSVHI   350

351   VEEIIDKFRRGEQDRAEFWINKPGVFIYIVYVAIRDADGRFRGVMEMMQDCTRIRSLEGSRTLLTWDEEQ   420

421   SPAQGSKESESDTAGEDGIRPDTKLKSLLQRYPQLMDDLPTISSKFTLLRSPMAKVILPVATIKMMSERA   517

491   DIPSDMLIGKLESLIASYNKPDRSEEK                                              517
```

SUPPLEMENTAL FIGURE 3 (A) Sequences of the linker segments between input (green) and GGDEF (blue) domains of selected diguanylate cyclases. The linker segment between the hemerythrin and PAS10 domains of PG_0686 is also included. A hydrophobic heptad repeat (yellow box) is largely found in the linker segments, usually resulting in a coiled-coil secondary structure. The leucine heptad repeat, characteristic of diguanylate cyclase linker sections, is observed in the PG_0686 protein sequence. **(B)** The protein sequence of PG_0686 showing the hemerythrin (BHr) (green box), PAS10 (blue box), and

DUF1858 (red box) domains. The binding residues as predicted by I-TASSER are also highlighted for the chloro diiron oxo ligand (blue residues) in the BHr domain and phosphate ligand (pink residues) in the PAS10 domain. The leucine repeats (navy blue) and cysteines (yellow) are also highlighted. The c-di-GMP inhibitory site motif, RxxD, is highlighted with orange residues.

A

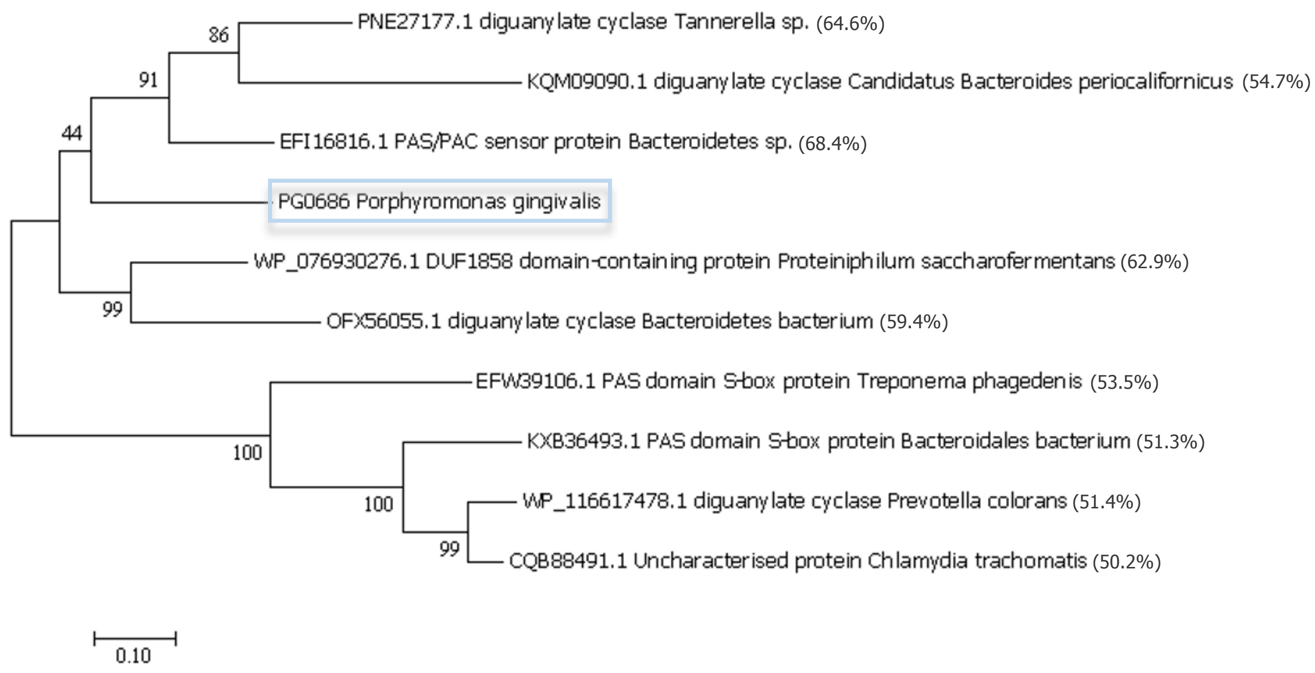

B

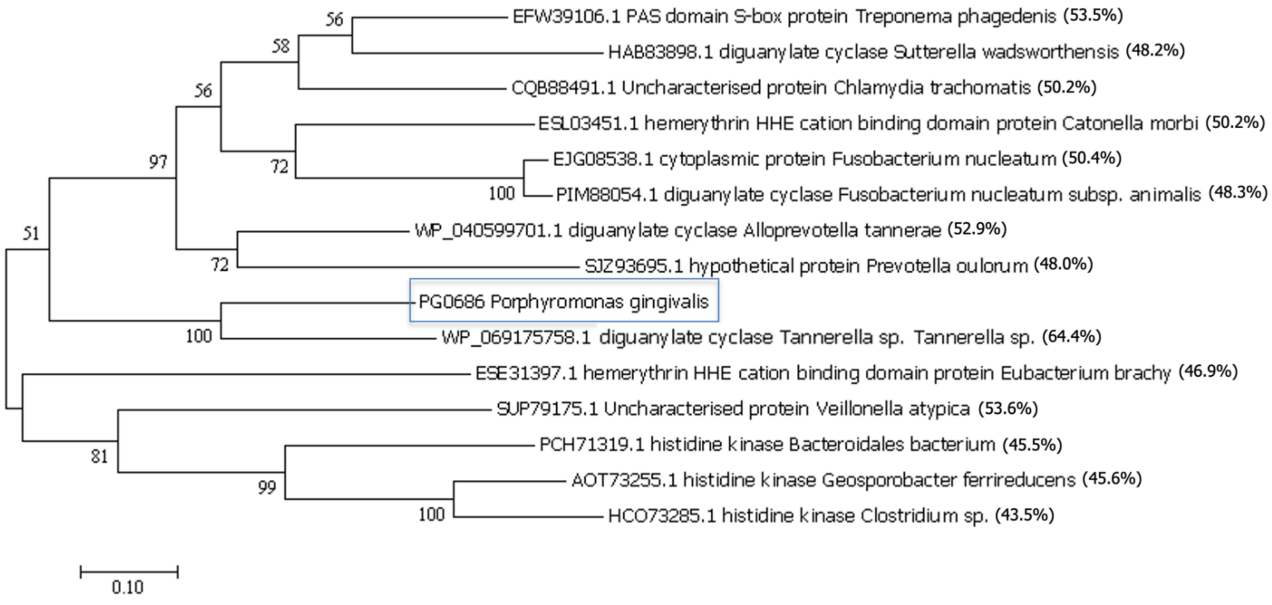

SUPPLEMENTAL FIGURE 4 A Molecular Phylogenetic analysis by Maximum Likelihood method of amino acid sequences obtained via a Blastp (NCBI database) using the PG_0686 sequence. The evolutionary history was inferred by using the Maximum Likelihood method based on the JTT matrix-based model. The tree with the highest log likelihood (-6602.91) is shown. The percentage of trees in which the

associated taxa clustered together is shown next to the branches. Initial tree(s) for the heuristic search were obtained automatically by applying Neighbor-Join and BioNJ algorithms to a matrix of pairwise distances estimated using a JTT model, and then selecting the topology with superior log likelihood value. The tree is drawn to scale, with branch lengths measured in the number of substitutions per site. The analysis involved 10 amino acid sequences. All positions containing gaps and missing data were eliminated. There were a total of 490 positions in the final dataset. Evolutionary analyses were conducted in MEGA7. Percentage values represent percentage identity of the proteins against PG_0686. **B** Molecular Phylogenetic analysis by Maximum Likelihood method of amino acid sequences obtained via a position specific iterative-BLAST (PSI-BLAST) (NCBI database) using the PG_0686 sequence. The evolutionary history was inferred by using the Maximum Likelihood method based on the JTT matrix-based model. The tree with the highest log likelihood (-7030.91) is shown. The percentage of trees in which the associated taxa clustered together is shown next to the branches. Initial tree(s) for the heuristic search were obtained automatically by applying Neighbor-Join and BioNJ algorithms to a matrix of pairwise distances estimated using a JTT model, and then selecting the topology with superior log likelihood value. The tree is drawn to scale, with branch lengths measured in the number of substitutions per site. The analysis involved 15 amino acid sequences. All positions containing gaps and missing data were eliminated. There were a total of 338 positions in the final dataset. Evolutionary analyses were conducted in MEGA7. Percentage values represent percentage identity of the proteins against PG_0686.

A

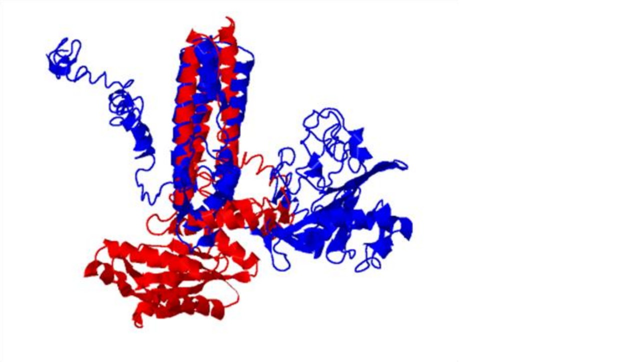

PG_0686 (blue) and VC1216 (red)
TM score = 0.19632, RMSD = 5.31

B

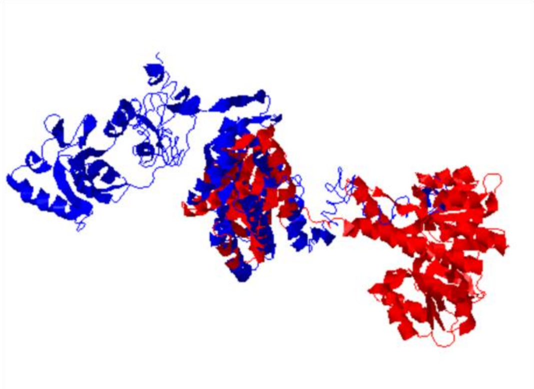

PG_0686 (blue) and PleD (red)
TM score = 0.21087, RMSD = 7.72

C

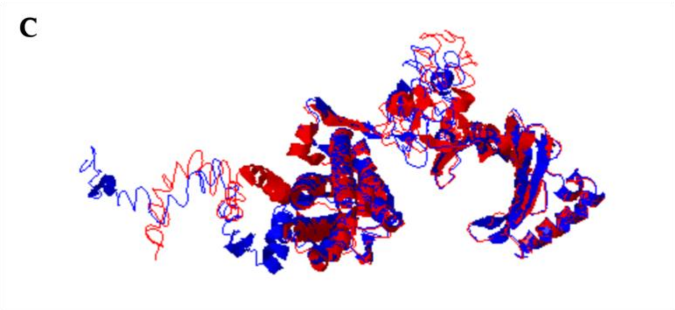

PG_0686 (blue) and WP_069175758.1
DGC *Tannerella* sp. (red)
TM score = 0.82718, RMSD = 3.46

SUPPLEMENTAL FIGURE 5 Structural similarity between PG_0686 with known diguanylate cyclase (DGC) proteins (A) VC1216 (*Vibrio cholerea*) and (B) PleD (*Caulobacter crescentus*). (C) Structural similarity between PG_0686 with annotated digunylate cyclase protein WP069175758.1. (*Tannerella* sp.). The protein model for each protein was predicted by I-TASSER, and alignment was performed using the TM-align server. The template modeling ™ scores for each alignment pair is shown. A TM score was given to each alignment to indicate the strength of the alignment and structural relatedness of the proteins. (0.00 < TM score < 0.30 represented random structural similarity; (0.50 < TM score < 01.00 represented about the same fold). In addition, an RMSD value was assigned to the alignment (as the value approaches zero, the more structurally similar the proteins are).

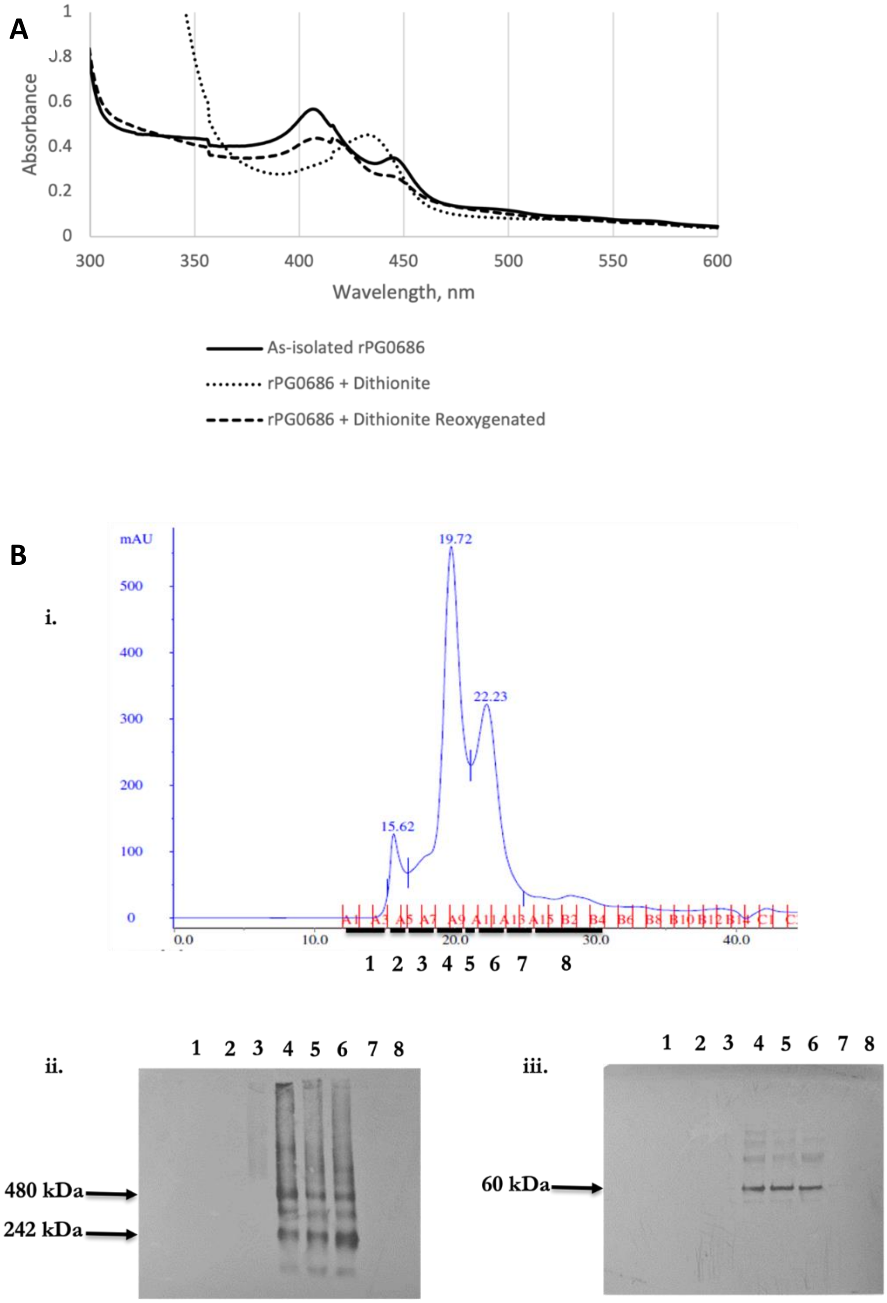
A
Absorbance
Wavelength, nm
As-isolated rPG0686
rPG0686 + Dithionite
rPG0686 + Dithionite Reoxygenated
B
i.
mAU
19.72
22.23
15.62
1 2 3 4 5 6 7 8
ii.
480 kDa
242 kDa
iii.
60 kDa

SUPPLEMENTAL FIGURE 6 Physical properties of rPG_0686 (A)UV-vis spectra absorption of rPG_0686 protein in 10 mM Tris-HCl (pH 7.5) (As-isolated rPG_0686). rPG_0686 was incubated with sodium dithionite under anaerobic conditions which resulted in the loss of the second peak and a spectrum shift to the right (rPG_0686 + Dithionite). The deoxygenated sample was left to reoxygenate overnight and the characteristic peaks of the rPG_0686 protein spectra were restored (rPG_0686 + Dithionite Reoxygenated). The rPG_0686 protein may reversibly bind oxygen. **(B)** Characterization of the rPG_0686 protein indicates that the protein is multimeric under native conditions. (i) Size exclusion chromatography (SEC) analysis of purified rPG_0686 showed three peaks which corresponded to sizes ca. 624 kDa (10mer) and 305 kDa (5mer), indicating that rPG_0686 is likely a multimeric protein under native conditions. (ii.) Immunoblot of a Native-PAGE gel of the indicated SEC fractions, confirming the multimeric conformation of rPG_0686 under native conditions. (iii.) Immunoblot of the corresponding SDS-PAGE gel which shows rPG_0686 in its monomeric 60 kDa form. Immunoblot analyses were done using polyclonal antibodies raised against purified rPG_0686 protein (Pierce).

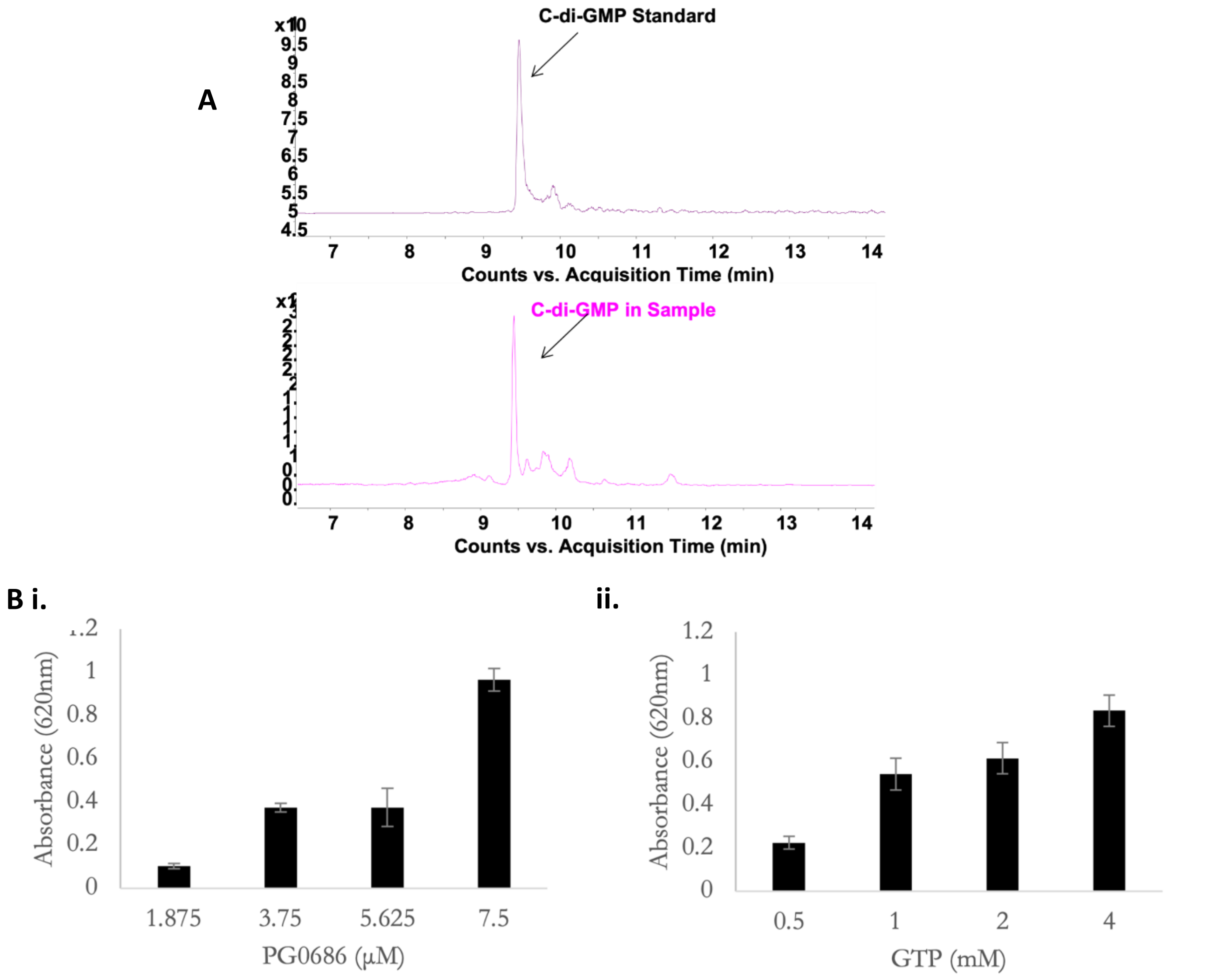

SUPPLEMENTAL FIGURE 7 C-di-GMP was present in *P. gingivalis* (A) LC-MS/MS confirmed the presence of c-di-GMP in the *P. gingivalis* samples, when c-di-GMP levels in the FLL361 mutant and W83 wild-type were measured. C-di-GMP levels in whole cell samples were determined and found to be reduced in FLL361. **(B)** Liberation of pyrophosphate increases when (i.) 0.5 mM GTP is in the presence of increasing concentrations of rPG_0686 or (ii.) 3.75 μM rPG_0686 is in the presence of increasing concentrations of GTP.

SUPPLEMENTAL TABLE 1 qRT-PCR validation of RNA-seq analysis for selected genes differentially expressed in FLL361/W83

		RNA-seq		qRT-PCR	
Gene ID	Annotation	Fold change	*P* value	Fold change	SD
Anaerobic					
PG_1551	Heme binding protein HmuY	9.76	4.60E-03	2.20	0.31
PG_1553	Cobaltochelatase	11.76	2.41E-02	5.15	0.73
PG_2212	Hypothetical protein PG_2212	9.14	4.56E-02	2.10	1.28
PG_0933	Elongation factor G	-6.10	5.36E-03	-26.87	0.27
PG_1030	T9SS C-terminal domain-containing protein	-7.57	3.26E-03	-9.15	4.78
PG_2102	T9SS C-terminal domain-containing protein	-6.86	5.22E-03	-14.92	0.03
H_2O_2 stress					
PG_0893	Hydroxylamine reductase	4.92	4.47E-02	6.43	0.26
PG_1181	TetR/AcrR family transcriptional regulator	8.52	3.13E-03	4.89	1.48
PG_1551	Heme binding protein HmuY	9.77	4.09E-03	12.29	1.51
PG_1030	T9SS C-terminal domain-containing protein	-7.57	1.85E-03	-8.28	0.94
PG_1271	Ornithine-oxo acid transaminase	-5.68	3.50E-03	-4.17	0.66
PG_1330	Large conductance mechanosensitive channel protein MscL	-5.01	3.33E-02	-6.93	2.79
PG_2102	T9SS C-terminal domain-containing protein	-4.94	1.10E-02	-2.85	0.07

SUPPLEMENTAL TABLE 2 Strains and plasmids used in this study

Strain or plasmid	Description	Reference or source
Strains		
Porphyromonas gingivalis		
W83	Wild type	Dou et al., 2010
FLL361	*ΔPG_0686::ermF*	This study
FLL361C'	FLL361 with pFLL361	This study
Escherichia coli		
BL21Star™ (DE3)pLysS	F- *ompT hsdS*B (rB-mB-) *gal dcm rne131* (DE3) pLysS (Cam^R)	Invitrogen
Top10	F- *mcr*A Δ(*mrr-hsd*RMS-*mcr*BC) φ80*lac*ZΔM15 Δ*lac*X74 *rec*A1 *ara* D139 Δ(*araleu*) 7697 *gal*U *gal*K *rps*L (StrR) *end*A1 *nuPG*	Invitrogen
DH5α	F– φ80*lac*ZΔM15 Δ(*lac*ZYA-*arg*F)U169 *rec*A1 *end*A1 *hsd*R17 (rK–, mK+) *pho*A *sup*E44 λ– *thi*-1 *gyr*A96 *rel*A1	Invitrogen
Plasmids		
pEXP5-NT/TOPO®	Ap^r, His tag	Invitrogen
pEXP5-NT-0686	Ap^r, His tag	This study
pFLL361	Ap^r, *tet*Q, pT-COW (*PG_0686*)	This study
pT-COW	Ap^r, *tet*Q	Gardener et al., 1996
pUC19	Ap^r	Invitrogen
pET11-PleD*His6	Ap^r, His tag	Kazmierczak lab

SUPPLEMENTAL TABLE 3 Primers used in this study

Use and name of primer	Nucleotide Sequence
Complemention	
SalI/*PG_0686* Fwd	5’ TGT CGA CCT TAA TCA TCT CTT TTC GAG GGA C 3’
SalI/*PG_0686* Rvs	5’ TGT CGA CCT ATT TCT CTT CCG ATC GAT CC 3’
Mutant Construction	
ErmF Fwd	5’ TGA CTA ACT AGG AGG AAT AAA TGA CAA AAA AGA AAT TGC CCG 3’
ErmF Rvs	5’ GAT TAT TCC CTC CAG GTA CTA CGA AGG ATG AAA TTT TTC A 3’
Upstream PG_*0686* Fwd	5’ CTT CGA AAA TGG AGC CAT CGC GAA A 3’
Upstream *PG_0686* Rvs	5’ TCA TTT ATT CCT AGT TAG TCA CAT GTT TTT TGA GCA ATT AGT GAT G 3’
Downstream *PG_0686* Fwd	5’ TTC GTA GTA CCT GGA GGG AAT AAT CTA GCA TGA AGA AGG AGG TGT GTC AAT 3’
Downstream *PG_0686* Rvs	5’ CTT GTC GCT CTT CCT TTC ACC TCA 3’
qPCR	
Internal *PG_0686* Fwd	5’ CAA TAA GCC CGG AGT CTT CA 3’
Internal *PG_0686* Rvs	5’ GCA GTA TCG GAT TCG CTT TC 3’
16S rRNA Fwd	5’ CGA TGA TTA CTA GGA GTT TGC GAT 3’
16S rRNA Rvs	5’ CAC CAT CCG TCA TCT ACA TTT CAA 3’
RNASeq Validation	
PG_0893 Rlt PCR Fwd	5’ CGG CTA CGT GGA TGA AGA GAT CAA 3’
PG_0893 Rlt PCR Rev	5’ CGA CAC CTA TAT TTA CTT CCG TGA TTT 3’
Fwd *PG_0933*_IP	5’ AGC CTG CCA TCT TCA TCA TCA TC 3’
Rvs *PG_0933*_IP	5’ GGC ACT ATC TTG GAG CCA TAT C 3’
Fwd *PG_1030*_IP	5’ TGG GAT TAT GCT CAG GAG TAA AG 3’
Rvs *PG_1030*_IP	5’ TTA TCT TCC CGG CCA AAG TAT AG 3’
PG_1181 Rlt PCR Fwd	5’ ATC TGC AGC TCT AAG AAA AAT ACC 3’
PG_1181 Rlt PCR Rev	5’ TAT CTG CAT CAG TGC TCG CC 3’
Fwd *PG_1271*_IP	5’ CCC TCT TTG TGG CTG ATG AA 3’
Rvs *PG_1271*_IP	5’ GCT TTA CCG AGC ACA ACA ATA TC 3’
Fwd *PG_1330*_IP	5’ AAA GAT CGT CAC CTC GTT GG 3’
Rvs *PG_1330*_IP	5’ GAC GAC AGC CTT GCT CAA TA 3’
Fwd *PG_1551*_IP	5’ CGC TAT GAC GTT CGT CTC AAT 3’
Rvs *PG_1551*_IP	5’ CGG TAG TAG CCT GAT CCA TTT C 3’
Fwd *PG_1553*_IP	5’ GGA GCA GGA CAA CGA TGA TAA A 3’
Rvs *PG_1553*_IP	5’ GTC AAG GAT GGA GTC GAG TTT C 3’
Fwd *PG_2102*_IP	5’ TGC TGG ACG AGG ATA ACA ATA C 3’
Rvs *PG_2102*_IP	5’ CCG AAT GTC CCA ATC CGA ATA 3’
PG_2212_realtime_F	5’ GAT ATC CGG GGC TCT CCT CTT CT 3’
PG_2212_realtime_R	5’ CAG AAC GAA AAA AAT CTC GCG CCA CTT 3’
RT-PCR	

Full *PG_0686* Fwd	5' ATG CAG GTC ATA AAA ACA AAT GAA 3'
Full *PG_0686* Rvs	5' TTT CTC TTC CGA TCG ATC CG 3'
Full 16S rRNA Fwd	5' AGG CAG CTT GCC ATA CTG CG 3'
Full 16S rRNA Rvs	5' ACT GTT AGC AAC TAC CGA TGT 3'
Full *PG_0686* Rvs with STOP	5' CTA TTT CTC TTC CGA TCG ATC C 3'
Sequencing	
T7 Fwd	5' TAA TAC GAC TCA CTA TAG GG 3'
T7 Rvs	5' TAG TTA TTG CTC AGC GGT GG 3'
Internal *PG_0686* PLUS	5' CCG GAA TTA GAT GCA CCG 3'
Internal *PG_0686* MINUS	5' CAT ACC ATG TTT GGC AAG TAA GG 3'

SUPPLEMENTAL FIGURE LEGENDS

SUPPLEMENTAL FIGURE 1 The creation of the FLL361 mutant. (A) PCR, amplification from chromosomal DNA, confirming the creation of the FLL361 mutant. The *PG_0686* gene was present in the *P. gingivalis* W83 wild-type but absent, and replaced by *ermF*, in the FLL361 mutant. **(B)** RT-PCR shows that PG_*0686* is present but not Induced under H_2O_2 stress in the complemented strain FLL361C'. A – cDNA from Untreated C361' culture; B – cDNA from Treated C361' culture; C – 16S r cDNA control from Untreated C361' culture; D – 16S r cDNA control from Treated C361' culture.

SUPPLEMENTAL FIGURE 2 Gene enrichment from RNA-seq data. (A) The top enriched pathways for differentially upregulated (i) and downregulated (ii) genes in *P. gingivalis* FLL361 under normal anaerobic conditions. **(B)** The top enriched pathways for differentially upregulated (i) and downregulated (ii) genes in *P. gingivalis* FLL361 under HPS conditions. Enrichment plots were generated by ShinyGO 0.76 (Ge, et al., 2020).

SUPPLEMENTAL FIGURE 3 (A) Sequences of the linker segments between input (green) and GGDEF (blue) domains of selected diguanylate cyclases. The linker segment between the hemerythrin and PAS10 domains of PG_0686 is also included. A hydrophobic heptad repeat (yellow box) is largely found in the linker segments, usually resulting in a coiled-coil secondary structure. The leucine heptad repeat, characteristic of diguanylate cyclase linker sections, is observed in the PG_0686 protein sequence. **(B)** The protein sequence of PG_0686 showing the hemerythrin (BHr) (green box), PAS10 (blue box), and DUF1858 (red box) domains. The binding residues as predicted by I-TASSER are also highlighted for the chloro diiron oxo ligand (blue residues) in the BHr domain and phosphate ligand (pink residues) in the PAS10 domain. The leucine repeats (navy blue) and cysteines (yellow) are also highlighted. The c-di-GMP inhibitory site motif, RxxD, is highlighted with orange residues.

SUPPLEMENTAL FIGURE 4 A Molecular Phylogenetic analysis by Maximum Likelihood method of amino acid sequences obtained via a Blastp (NCBI database) using the PG_0686 sequence. The evolutionary history was inferred by using the Maximum Likelihood method based on the

JTT matrix-based model. The tree with the highest log likelihood (-6602.91) is shown. The percentage of trees in which the associated taxa clustered together is shown next to the branches. Initial tree(s) for the heuristic search were obtained automatically by applying Neighbor-Join and BioNJ algorithms to a matrix of pairwise distances estimated using a JTT model, and then selecting the topology with superior log likelihood value. The tree is drawn to scale, with branch lengths measured in the number of substitutions per site. The analysis involved 10 amino acid sequences. All positions containing gaps and missing data were eliminated. There were a total of 490 positions in the final dataset. Evolutionary analyses were conducted in MEGA7. Percentage values represent percentage identity of the proteins against PG_0686. **B** Molecular Phylogenetic analysis by Maximum Likelihood method of amino acid sequences obtained via a position specific iterative-BLAST (PSI-BLAST) (NCBI database) using the PG_0686 sequence. The evolutionary history was inferred by using the Maximum Likelihood method based on the JTT matrix-based model. The tree with the highest log likelihood (-7030.91) is shown. The percentage of trees in which the associated taxa clustered together is shown next to the branches. Initial tree(s) for the heuristic search were obtained automatically by applying Neighbor-Join and BioNJ algorithms to a matrix of pairwise distances estimated using a JTT model, and then selecting the topology with superior log likelihood value. The tree is drawn to scale, with branch lengths measured in the number of substitutions per site. The analysis involved 15 amino acid sequences. All positions containing gaps and missing data were eliminated. There were a total of 338 positions in the final dataset. Evolutionary analyses were conducted in MEGA7. Percentage values represent percentage identity of the proteins against PG_0686.

SUPPLEMENTAL FIGURE 5 Structural similarity between PG_0686 with known diguanylate cyclase (DGC) proteins (A) VC1216 (*Vibrio cholerea*) and (B) PleD (*Caulobacter crescentus*). (C) Structural similarity between PG_0686 with annotated digunylate cyclase protein WP069175758.1. (*Tannerella* sp.). The protein model for each protein was predicted by I-TASSER, and alignment was performed using the TM-align server. The template modeling ™ scores for each alignment pair is shown. A TM score was given to each alignment to indicate the strength of the alignment and structural relatedness of the proteins. (0.00 < TM score < 0.30 represented random structural similarity; (0.50 < TM score < 01.00 represented about the same fold). In addition, an RMSD value was assigned to the alignment (as the value approaches zero, the more structurally similar the proteins are).

SUPPLEMENTAL FIGURE 6 Physical properties of rPG_0686 (A)UV-vis spectra absorption of rPG_0686 protein in 10 mM Tris-HCl (pH 7.5) (As-isolated rPG_0686). rPG_0686 was incubated with sodium dithionite under anaerobic conditions which resulted in the loss of the second peak and a spectrum shift to the right (rPG_0686 + Dithionite). The deoxygenated sample was left to reoxygenate overnight and the characteristic peaks of the rPG_0686 protein spectra were restored (rPG_0686 + Dithionite Reoxygenated). The rPG_0686 protein may reversibly bind oxygen. **(B)** Characterization of the rPG_0686 protein indicates that the protein is multimeric under native conditions. (i) Size exclusion chromatography (SEC) analysis of purified rPG_0686 showed three peaks which corresponded to sizes ca. 624 kDa (10mer) and 305 kDa (5mer), indicating that rPG_0686 is likely a multimeric protein under native conditions. (ii.)

Immunoblot of a Native-PAGE gel of the indicated SEC fractions, confirming the multimeric conformation of rPG_0686 under native conditions. (iii.) Immunoblot of the corresponding SDS-PAGE gel which shows rPG_0686 in its monomeric 60 kDa form. Immunoblot analyses were done using polyclonal antibodies raised against purified rPG_0686 protein (Pierce).

SUPPLEMENTAL FIGURE 7 C-di-GMP was present in *P. gingivalis* (A) LC-MS/MS confirmed the presence of c-di-GMP in the *P. gingivalis* samples, when c-di-GMP levels in the FLL361 mutant and W83 wild-type were measured. C-di-GMP levels in whole cell samples were determined and found to be reduced in FLL361. **(B)** Liberation of pyrophosphate increases when (i.) 0.5 mM GTP is in the presence of increasing concentrations of rPG_0686 or (ii.) 3.75 μM rPG_0686 is in the presence of increasing concentrations of GTP.
